# The Health-Related and Learning Performance Effects of Air Pollution and Other Urban-Related Environmental Factors on School-Age Children and Adolescents—A Scoping Review of Systematic Reviews

**DOI:** 10.1007/s40572-024-00431-0

**Published:** 2024-02-19

**Authors:** Inés Valls Roche, Mònica Ubalde-Lopez, Carolyn Daher, Mark Nieuwenhuijsen, Mireia Gascon

**Affiliations:** 1 ISGlobal, Parc de Recerca Biomèdica de Barcelona–PRBB, C/ Doctor Aiguader, 88, 08003 Barcelona, Spain; 2 Universitat Pompeu Fabra (UPF), Barcelona, Spain; 3 CIBER Epidemiología y Salud Pública (CIBERESP), Madrid, Spain

**Keywords:** Air pollution, Green space, Children, Adolescents, Health, School

## Abstract

**Purpose of Review:**

This scoping review aims to assess the impact of air pollution, traffic noise, heat, and green and blue space exposures on the physical and cognitive development of school-age children and adolescents. While existing evidence indicates adverse effects of transport-related exposures on their health, a comprehensive scoping review is necessary to consolidate findings on various urban environmental exposures’ effects on children’s development.

**Recent Findings:**

There is consistent evidence on how air pollution negatively affects children’s cognitive and respiratory health and learning performance, increasing their susceptibility to diseases in their adult life. Scientific evidence on heat and traffic noise, while less researched, indicates that they negatively affect children’s health. On the contrary, green space exposure seems to benefit or mitigate these adverse effects, suggesting a potential strategy to promote children’s cognitive and physical development in urban settings.

**Summary:**

This review underscores the substantial impact of urban exposures on the physical and mental development of children and adolescents. It highlights adverse health effects that can extend into adulthood, affecting academic opportunities and well-being beyond health. While acknowledging the necessity for more research on the mechanisms of air pollution effects and associations with heat and noise exposure, the review advocates prioritizing policy changes and urban planning interventions. This includes minimizing air pollution and traffic noise while enhancing urban vegetation, particularly in school environments, to ensure the healthy development of children and promote lifelong health.

**Supplementary Information:**

The online version contains supplementary material available at 10.1007/s40572-024-00431-0.

## Introduction

In the last decades, cities have increasingly relied on motor vehicles as a means of transportation, which has often led to ignoring active and public transportation as alternatives [1]. This dependence on private motorized transport has led to a lack of or inadequate access to green spaces, and high levels of harmful environmental exposures, such as air pollution, traffic noise, and increased urban heat, which can lead to sedentary behaviour and risk for respiratory and cardiovascular diseases, among others [2–4].

Air pollution is a harmful environmental exposure, responsible for approximately 7 million deaths annually [5]. Around 93% of the world’s children under 15 years old breathe air so polluted that it affects their health [6–8]. Another urban environmental threat is noise exposure, which has become unavoidable due to growing transport demand and is considered the second most significant environmental cause of ill health in Western Europe [9]. Exposure to environmental noise in moderate doses may have a wide range of non-auditory effects in children and adults [10]. Similarly, increasing heat and the related urban heat island effect (UHI) is another environmental concern in urban areas, which describes a phenomenon in which urban areas commonly have a higher temperature of up to 12° at night than the surrounding rural areas [11].

The younger generations are more sensitive and at increased risk when exposed due to their current state of cognitive and physical development. Air pollution can damage the developing brain and impair cognitive function across the lifespan. Moreover, children and adolescents are more susceptible to being exposed to traffic noise as, due to their cognitive development, they have fewer coping repertoires than adults to deal with and have control over noise [12]. On the contrary, exposure to green and blue spaces is associated with positive effects on children’s and adolescents’ physical and mental development, such as stress relief, increased social connectivity, and physical activity [13–18].

However, existing literature reviews primarily focus on specific urban environmental exposures and their isolated health impacts on children [19, 20]. Currently, there is still an absence of a synthesis of joint evidence on how air pollution, noise, heat, and green and blue spaces affect children’s and adolescents’ health and learning performance, especially in the school environment, as children spend considerable time in these settings [21]. This gap in the research field impedes decision-makers from acting on and promoting evidence-based interventions and policies to ensure healthy development among children and adolescents.

## Research Aims and Objectives

The research questions the present review seeks to answer are as follows: “What are the impacts of urban air pollution, heat, and traffic noise exposures on the cognitive and physical development and learning performance of school-age children and adolescents? And what are the effects of urban green and blue space exposures on the cognitive and physical development and learning performance of children and adolescents?” The research questions were addressed through a scoping review of current systematic reviews and meta-analyses.

## Methods

We conducted a scoping review of systematic reviews (with or without meta-analyses) and meta-analyses. According to the Canadian Institute of Health Research, scoping reviews are “exploratory projects that systematically map the literature available on a topic, identifying key concepts, theories, sources of evidence and gaps in the research” [22]. Scoping reviews are typically used when the topic is broad, and there is a large volume of literature available [23], such as this case.

This scoping review followed the framework proposed by Peters et al. and followed the reporting method by the Preferred Reporting Items for Systematic Reviews and Meta-analysis extension for Scoping Reviews (PRISMA-ScR) [24, 25]. The PRISMA-ScR checklist is composed of several steps, which are (1) eligibility criteria, (2) information sources, (3) search, (4) selections of sources of evidence, (5) data charting process and data items, (6) critical appraisal of individual sources of evidence, and (7) synthesis of results. The PRISMA-ScR checklist can be consulted in the supplementary material.

### Eligibility Criteria

The eligibility criteria for the data selection of the articles are the following:

- The study design should be a systematic review (with or without meta-analyses) or a meta-analysis. In regards to the type of study design of the studies included in the reviews, no restrictions were applied.
- The reviews include primary research studies that investigate the long-term and/or short-term effects of the previously mentioned urban-related environmental exposures on children’s and adolescents’ health (i.e., cognitive health, mental health, behavioural outcomes (Autism Spectrum Disorder (ASD), Attention deficit hyperactivity disorder (ADHD), Hyperactivity), respiratory health, cardiovascular health, and related risk factors), and learning performance, while also looking at the physical activity (PA) as a potential mediator for that relationships.
- The study population included in the reviewed studies should be school-age children and adolescents (from 5 to 18 years old), regardless of sex, gender, health status, and geographic location. These studies can include other population (e.g., infants or adults); however, these populations should be analysed separately from the ones of interest for our review.
- The papers should be written in English or Spanish.
- The systematic reviews and meta-analysis must be published between 2017 and 2023 as we are including systematic reviews and meta-analysis that summarize previous studies; we aim to find the most updated evidence on this field of research.

### Information Sources and Search

We searched for systematic reviews and meta-analysis on the 11th of May 2023 on two electronic databases: PubMed and ScienceDirect. We used a series of keywords and terms related to the environmental exposures, health-related outcomes of interest, learning performance, and physical activity outcomes. To ensure all key terms and MeSH terms were broad enough to cover any related literature and prevent chances of relevant information being overlooked, PubMed’s MeSH Term tool was used to detect all MeSH terms available, more information on the search strategy in the Appendix B (see Supplementary Material). On PubMed, we applied the restrictions of population (child: 0–18 years), study design (systematic reviews and meta-analysis), and year of publication (2017–2023).

### Selection of Sources of Evidence

All articles found through the search strings were retrieved in the Rayyan software to be screened for title and abstract against the criteria selection [26]. Afterwards, the full text of potentially eligible systematic reviews and meta-analysis was assessed comprehensively following the inclusion criteria.

### Data Charting Process

The data extracted from the final included reviews were entered into a database, Microsoft Excel 2016, so that the following data could be extracted and charted according to the variables of interest:

- Author(s) and year of publication
- Exposure(s) assessed
- Outcome(s) assessed
- Number and type of studies included in the review and, if applicable, the date range of the studies published
- Sample size and age range of the study population
- Countries in which the original studies were conducted
- Effect size (e.g. odds ratio (OR) and confidence interval in a meta-analysis)
- Method for assessing quality and risk of bias (if used any) of the studies included
- Quality assessment results
- Main findings
- Relevant limitations of the review that may influence the interpretation of the findings

### Critical Appraisal of Individual Sources of Evidence

In this review, a critical appraisal was not conducted. However, we have emphasized throughout the results section that certain associations between exposures and health outcomes included meta-analysis, which results in a stronger and more accurate evidence. Also, this step in the framework by Peters et al. and PRISMA-ScR is optional, and it is largely dependent on the purpose of the review and subject to the author’s judgement [24, 25]. Nonetheless, we determined the strength of evidence of a causal relationship based on an adapted version of the guidelines for the level of evidence employed by the International Agency for Research on Cancer [27] that has been used in earlier studies in this field of research [28] (Table 2).

### Synthesis of Results

We grouped the reviews and meta-analysis in the “Findings” section by the environmental exposures assessed and then for the health and learning performance outcomes measured. Associations, statistically significant and non-significant, between exposure and outcome that were identified were represented within each results subsection.

## Findings

### Literature Search Results

**Fig. 1:**
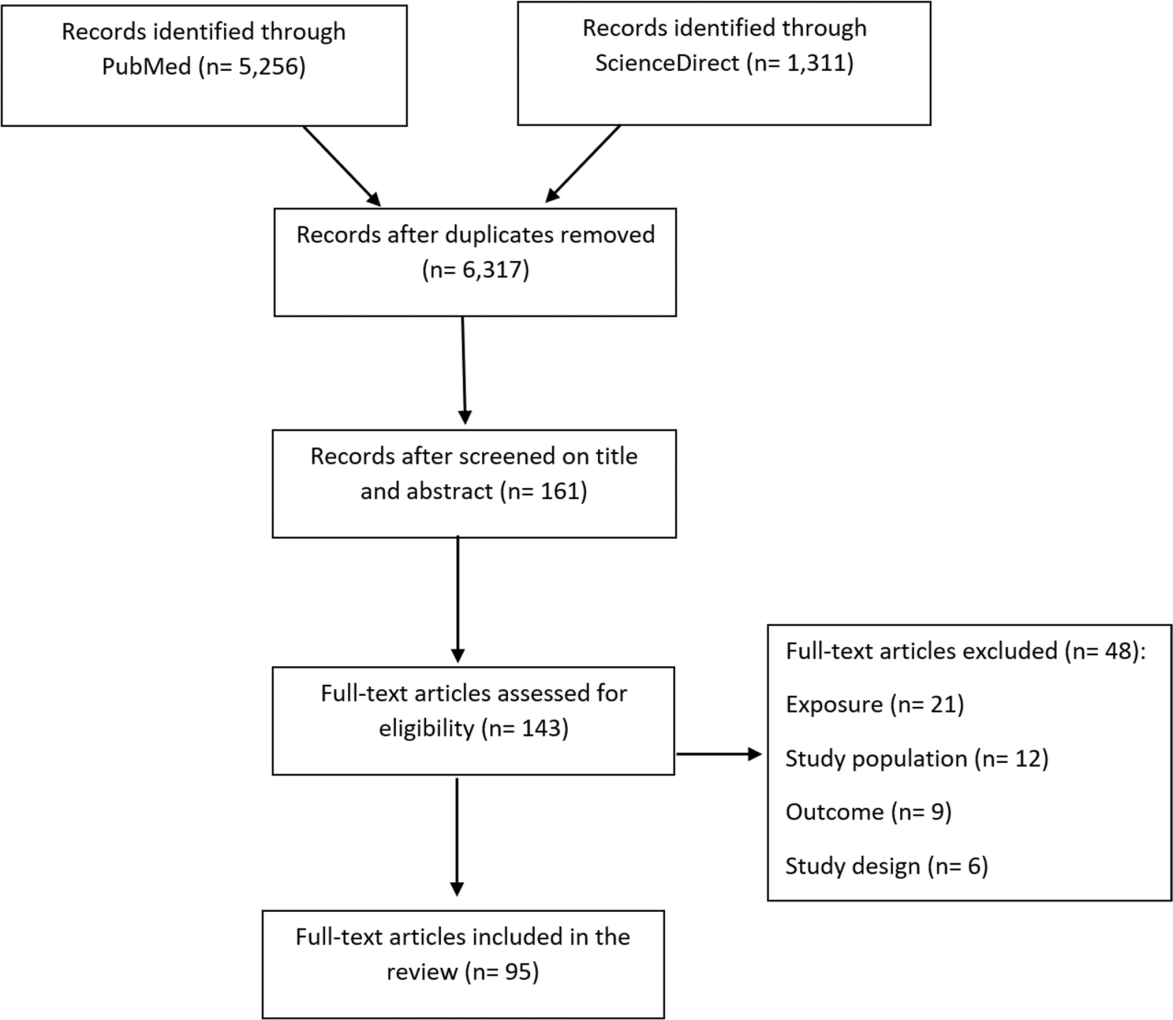
A structured summary of the research process undertaken on the 11th of May 2023

The literature search (11 May 2023) in the two electronic databases (ScienceDirect and PubMed) identified a total of 6317 records. After screening the titles and abstracts, the total number of studies that fit the selection criteria of being a systematic review and/or meta-analysis was 161, all published between 2017 and 2023, and that analysed exposures and outcome(s) relevant for this review, including our target population. After screening full-text and extracting the relevant information into the table, the number of studies was 95 systematic reviews and meta-analyses that were included in this review. The flow chart of the scope review search is summarized in Fig. 1, and the search strategy can be found in Appendix B (Supplementary Material).

### General Findings

Ninety-five systematic reviews (with or without meta-analyses) and meta-analyses were included in this scope review, all of which were analysed in detail in the Appendix C (see Supplementary Material). Beyond children and adolescents, some reviews included prenatal and postnatal periods of exposure, adults and elderly populations, yet investigated separately. Thus, for the focus of this review, solely the evidence on children and adolescent populations was evaluated. The findings are divided into the different urban exposures and its effects on cognitive, behavioural, learning and mental health outcomes, respiratory health outcomes, and cardiovascular health outcomes. Traffic-related air pollution (TRAP) is composed of several air pollutants (i.e. particulate matter (PM), nitrogen dioxide (NO_2_), carbon dioxide (CO_2_), ozone (O_3_), black carbon (BC), elemental carbon (EC), polycyclic aromatic hydrocarbons (PAHs), and heavy metals); however, because some of the reviews investigated the health effects associated with each air pollutant individually, the findings have been organized according to each environmental exposure and health outcome evaluated, as detailed in Table 1.

**Table 1:** Number of studies included for each environmental exposure and health outcome

Exposure	Total *n* of studies	Cognitive, behavioural, and mental health outcomes	Respiratory health outcomes	Cardiovascular health outcomes and related risk factors
TRAP	25	11	12	5
PM	24	6	12	7
NO2	10	0	7	3
PAHs	2	1	1	0
O3	6	0	4	2
EC/BC	2	1	0	1
Metals	4	3	0	1
Noise	6	4	0	1
Heat	5	0	1	1
Green spaces	25	18	7	8
Blue Spaces	1	1	0	1

### Traffic-Related Air Pollution (TRAP)

#### Cognitive, Behavioural, Learning and Mental Health Outcomes

The evidence has shown that exposure to traffic-related air pollution may damage the developing brain and the central nervous system (CNS) in various ways [29]. Specifically, TRAP exposure is associated with decreased mental and/or psychomotor development [30], behavioural disorders, ASD prevalence and development [29], decreases in cognitive function, and increased neuroinflammatory markers [29, 31, 32]. Furthermore, two reviews suggest that air pollution exposure is associated with alterations in the brain structure, function, and metabolism [33]; however, future studies are needed to confirm this [34].

Short- and long-term TRAP exposure in and around school and home is significantly associated with poorer academic performance scores [32, 35], impairments in problem-solving abilities, lower grade point average (GPA), and adversely affected executive function, with the effect becoming stronger over time being exposed [36]. This is worrisome, as while school-age children spend 4–5% of recording time commuting, they receive around 12 to 20% and 10 to 11% of their daily BC and UFP exposure, respectively [37, 38].

In relation to mental health, articles suggest that adolescents’ exposure to TRAP was significantly associated with symptoms of depression [33], generalized anxiety disorder, psychotic disorders, delusions, hallucinations, unusual experiences, and poorer general mental health [32, 38, 39].

#### Respiratory Health Outcomes

The following reviews suggest that TRAP exposure influences the respiratory health of children and adolescents in numerous ways. TRAP short- and long-term exposure has been negatively associated with small airway infections in children—with a more notable effect on boys—[40], increases in paediatric pneumonia-related hospitalizations (OR = 1.8%, 95% CI: 0.5–3.1%) [41], low FEV_1_ [42], high prevalence of childhood asthma in Latin America countries and the Caribbean [43], and is considered a risk factor for allergic rhinitis (AR). Specifically, children and adolescents exposed to higher concentrations of air pollution were 36% more likely to experience AR than those less exposed in South American countries [44] and 89% and 63% more likely in Asian and European countries, respectively [45]. Additionally, even if they become less exposed during adulthood, they are still more likely to present decreased lung function and increased risk for respiratory diseases due to previous childhood exposure [46, 47]. Moreover, high TRAP concentrations at school increase the risk of suffering bronchitis, bronchopneumonia, and having lower pulmonary function parameters compared to children attending schools with lower TRAP levels [36].

Conversely, Fuertes et al.’s meta-analysis of five European birth cohorts found no evidence to support that long-term exposure to air pollution at home increased the prevalence of rhinoconjunctivitis, asthma, or paediatric eczema [48]. Lastly, Lam et al. reported that the interactions between airborne allergens and air pollutants influenced severe asthma symptoms requiring hospitalization [49].

#### Cardiovascular Health Outcomes and Related Risk Factors

The retrieved reviews show that TRAP exposure during childhood and adolescence may impact cardiovascular health. For instance, TRAP exposure has been significantly correlated with high blood pressure (BP) in children [50], rapid weight gain, or higher body mass index (BMI) [51, 52] and has been linked to increased acute morbidity and mortality of cardiovascular diseases (CVDs) [36]. Lastly, a review found that when children and adolescents exercised in highly polluted areas, the reported benefits of PA on cardiopulmonary fitness were null, and it even had detrimental health effects due to breathing polluted air, such as a decrease in glucose resistance and higher risk of asthma development [53].

### Particulate Matter

#### Cognitive, Behavioural, Learning and Mental Health Outcomes

Particulate matter (PM) of different sizes is the most investigated air pollutant in the included reviews, and while some of these reviews examined PM_2.5_, PM_10_, and PM_1_ separately, many have investigated them concurrently. Thus, this review presents the results of PM_2.5_, PM_10_, and PM_1_ jointly while making a distinction when relevant.

PM exposure has been found to be associated with an increased risk of childhood ASD [54] and an increased risk of ADHD [55], with a more significant effect in boys than girls [54]. Moreover, other reviews suggest that [53] PM exposure is linked to attention deficits [55, 56]; specifically, PM_2.5_ was a risk factor for attention/executive functions at ages 6 to 11, especially for girls [56]. PM_2.5_ exposure was also associated with decreased learning and memory function and a higher risk of developing learning difficulties in boys. Moreover, an article found evidence that PM_2.5_ was detrimental to executive function skills, and PM_2.5_ during commuting has been associated with reduced growth in working memory [57]. Indeed, PM_2.5_ appears to be the most significant air pollutant associated with adverse CNS outcomes [29] and has the most detrimental effects compared to other air pollutants, such as NO_2_ and O_3_ [35].

#### Respiratory Health Outcomes

Regarding respiratory outcomes, PM exposure during childhood and adolescence is suggested to negatively affect the respiratory system of children and be associated with allergies, such as allergic rhinitis and asthma. Indeed, Khreis et al. estimated that compliance with the PM_2.5_ WHO Air Quality guideline values (annual mean of 10 μg/m3 at the time of the Khreis et al.’s review) could prevent 66,567 new cases of childhood asthma per year in Europe, and meeting the minimum levels of PM_2.5_ could prevent 191,883 (33% of all new cases per year) new cases of childhood asthma yearly [58].

Moreover, prolonged exposure to PM during childhood and PM_10_ high concentrations near home increased the risk of asthma-related emergency department visits and hospitalizations, increased readmission rates of childhood asthma, asthma exacerbations with high PM2.5 exposure [59], and was associated with wheezing episodes in children of 2–10 years of age [60, 61]. Luong et al. reported that exposure to PM_1_ was significantly associated with wheeze-associated disorders in Southeast Asia, with PM_1_ having more substantial effects [62]. Indeed, short- and long-term PM_1_ exposure was associated with decreased lung function [63], while PM_10_ was linked to a reduction in forced expiratory volume in 1 s (FEV1) [60]. Moreover, a meta-analysis found significant statistical associations between PM_2.5_ and PM_10_ exposure and the prevalence of childhood allergic rhinitis (OR = 1.09, 95% CI: 1.01–1.17; OR = 1.06, 95% CI: 1.02–1.11, respectively) [45]. Exposure to PM_10_ has been significantly associated with upper respiratory tract infections (URTI)-related emergency department visits and hospital admissions and outpatient visits, while PM_2.5_ was only significant to outpatient visits for URTI [64].

Furthermore, two reviews have reported that the strongest predictor of COPD in adulthood is childhood asthma; thus, the adverse health effects children have suffered during childhood can lead to irreversible lifelong changes, such as impaired lung function [65].

#### Cardiovascular Health Outcomes and Related Risk Factors

As the evidence shows, short- and long-term exposure to PM significantly contributes to cardiovascular toxicity and increased risk of CVD development. Several articles have reported a significant association between PM_2.5_ exposure and children’s elevated blood pressure (BP) [50, 66–68], which is also affected by both short- and long-term PM_10_ exposure [66, 67]. Long-term exposure to PM_10_ is associated with an increased risk of hypertension [67], as well as exposure to PM_1_ and PM_2.5_ [68], which they all linked to higher odds of childhood obesity and increased BMI [69, 70] Specifically, Huang et al. observed 12%, 28%, and 41% more odds of being obese in children more exposed to PM_10_, PM_2.5_, and PM_1_, respectively, than in those less exposed.

### Nitrogen Dioxide (NO_2_)

The second most examined air pollutant in the reviews included is nitrogen dioxide (NO_2_), which has been linked to poorer neurodevelopment, cardiovascular health, and respiratory outcomes. Ten reviews have investigated NO_2_ and these health outcomes.

#### Respiratory Health Outcomes

Evidence shows significant associations between NO_2_ exposure and increased asthma development in children [71], increased risk of asthma-related exacerbations in children, and asthma-related emergency department visits [72]. Specifically, for each 10 μg/m^3^ increase of NO_2_ exposure the odds of childhood asthma onset increased 5% and the odds of acute LRTIs onset of a 9% [73••]. NO_2_ exposure is also associated with the development of atopy, current wheezing, lower FEV_1_, and airflow obstruction in children with asthma attending schools with high NO_2_ concentrations [60, 72, 74].

#### Cardiovascular Health Outcomes and Related Risk Factors

Short- and long-term childhood exposure to NO_2_ is associated with high BP [75] and increased prevalence or risk of hypertension in children and adolescents [68] and is significantly associated with risk of childhood obesity and higher BMI [75]. Specifically, the odds increased by 12% (95% CI: 1.06–1.18) when being obese and exposed to high concentrations of NO_2_ than in less exposed children.

### Polycyclic Aromatic Hydrocarbons (PAHs)

#### Cognitive, Behavioural, Learning and Mental Health Outcomes

Children’s exposure to PAHs is reported to increase 35% the likelihood of negatively impacting children’s cognitive abilities, and the results show that among the gases examined (e.g. NO_2_, PAHs, and tobacco smoke), the most detrimental to children’s cognitive health were PAHs [76].

#### Respiratory Health Outcomes

Oliveira et al. stated that there is strong evidence that children attending schools from urban areas are being exposed to higher concentrations of PAHs than in rural areas, and high PAH levels at school have been associated with increased risk for asthma, pulmonary infections, increased carcinogenic risks, and allergies [77]. However, only one review documented such associations.

### Ozone (O_3_)

#### Respiratory Health Outcomes

Both long- and short-term exposure to O_3_ is linked to adverse pulmonary health effects, such as decreased peak expiratory flow (PEF) [74], low FEV_1_ in children with asthma, increased asthma-related visits [60], increased risks of asthma-related exacerbations [72], and childhood readmission rates [59].

#### Cardiovascular Health Outcomes and Related Risk Factors

Long-term exposure to O_3_ is significantly positively associated with elevated blood pressure [66], and increases of 10 μg/m^3^ in O3 exposure are linked to an increased risk of obesity [70].

### Black Carbon (BC) and Elemental Carbon (EC)

BC has been associated with detriments in executive function skills, especially for working memory [57]. Elemental carbon exposure at school is positively associated with overweight and obesity in school-age children [70].

### Metals

Heavy metals including lead (Pb), mercury (Hg), cadmium (Cd), aluminium (Al), copper (Cu), chromium (Cr), and barium (Ba) can be found in water, air, and soil, and animals such as fish [78, 79]. This review only included evidence regarding exposure to heavy metals that are found in the air and that can potentially affect children’s and adolescents’ health.

#### Cognitive, Behavioural, Learning and Mental Health Outcomes

Excessive metal exposure has a detrimental effect on the nervous system. Neurons and glia in the developing brain are vulnerable to the damage caused by metals such as lead and mercury, which may result in permanent neurodevelopmental damage [80].

Lead is the most well-known metal to affect cognitive health in children and behavioural disorders [81]. High levels of lead exposure are associated with higher odds of having ADHD [81, 82], loss of brain volume in the prefrontal cortex, and lower levels of gray matter [81] and have been referenced as one of the causes of ASD development [80].

Environmental mercury exposure is shown to increase the odds of ASD and ADHD [81], and this exposure can lead to neuroinflammation, dendritic overgrowth, and mitochondrial dysfunction.

#### Cardiovascular Health Outcomes and Related Risk Factors

Sanders et al. found significant associations between lead, inorganic arsenic, and cadmium exposure and childhood high blood pressure [50].

### Noise Exposure

#### Cognitive, Behavioural, Learning and Mental Health Outcomes

Road traffic noise at home and in school settings is significantly associated with hyperactivity or inattention problems and conduct problems [83–85]. However, in Clark and Paunovic’s review, the results did not suggest any effect, probably due to the low quality of the studies included [86]. According to Thompson et al.’s findings, lower noise exposure at school led to better reading and math scores, with 0.80 points higher in reading tests among students in quieter classrooms compared to those in noisier ones [87]. Also, low-quality studies suggested that noise exposure affected negatively academic performance. No significant associations were reported between noise exposure at school and executive function and reading or language abilities. In addition, a meta-analysis of two birth cohorts found no association between residential road traffic noise and emotional, aggressive, and ADHD-related symptoms in children [88].

#### Cardiovascular Health Outcomes and Related Risk Factors

Non-significant yet positive associations were found between road traffic noise in the kindergarten and high blood pressure [89].

### Heat Exposure

While heat exposure effects on children and adolescents have been less systematically explored compared to air pollution and noise, five reviews were included that were considered relevant. Three out of the five reviews documented the physiological and morphological differences in children compared to adults that make them more vulnerable to climate fluctuations caused by climate change [89–91]. Regarding the other two reviews, Cicco et al. reported that increased temperature associated with climate change may contribute to the prevalence of allergic rhinitis symptoms in children and to the increase in asthma-related emergency department visits [92]. The remaining review observed a U-shaped relationship in most included studies between PA and temperature, where children’s PA levels increased with temperatures up to 20–25 °C and then declined with higher temperatures [93].

### Green Exposure

Twenty-five reviews are included in this paper that evaluated the evidence on green space exposure and cognitive, respiratory, and cardiovascular health-related effects during childhood. While we aimed to include evidence on blue space exposure, three systematic reviews were included [94–96], but only one reported an association between blue exposure and health [96].

#### Cognitive, Behavioural, Learning and Mental Health Outcomes

Children’s exposure to green spaces, natural habitats, wooden playgrounds, and gardens has been linked to stress relief, improved focus, positive mood, improved well-being among children [97–101], and lower prevalence of depression and anxiety [102**•**, 103**•**]. It also contributes to children’s ability to establish relationships, improve behaviour, social competencies, and emotion management, and a decrease in internalizing/externalizing problems and peer-relationship problems [100, 104–108]. Lastly, studies by Mygind et al. suggest a positive impact of green exposure on cognitive abilities, motor skills, language, and communication skills [104]. Moreover, limited studies indicate that exposure to blue spaces, such as beaches, is positively associated with children’s emotional well-being, lower emotional problems, prosocial behaviour, and stress relief [96].

Furthermore, evidence indicates that green space exposure may reduce symptoms of ADHD, such as inattention and hyperactivity [97, 99, 106, 107, 109], and ASD [110]. Li et al. additionally reported a protective effect against schizophrenia [110]. Despite the emphasis on the health-related effects of green exposure in childhood, Li et al. highlighted that individuals with less childhood contact with nature experienced greater benefits from nature visits during adulthood [110].

Children residing in green surroundings exhibit larger tissue volumes in various brain regions, improvements in cognitive development, higher scores in self-discipline and intelligence, higher academic performance, and better spatial working memory scores than those with less greenery in their homes [99, 106]. Luque-García and colleagues found that children attending schools surrounded by more greenness showed superior working memory scores, academic performance, well-being, intelligence, cognitive development, and fewer ADHD symptoms compared to students at schools with less greenness [94, 95, 99]. Notably, these associations remained significant even after adjusting for socioeconomic characteristics, indicating that these effects remained significant regardless of the child’s socioeconomic background [99].

Moreover, four systematic reviews investigated interventions in green spaces, such as parks and playgrounds, and their impact on mental health, physical activity, and other outcomes. Becker et al. reported improved academic performance, learning motivation, social competence, positive behaviour patterns, and positive attitudes towards outdoor classes, with greater benefit for boy’s mental health in terms of physical activity [111]. Mnich et al. reported positive effects on attention and antisocial interactions when performing PA in green environments compared to non-green environments, as well as higher quality of life [112]. Moreover, Buckzyslowka et al. noted that a 20-30-min walk improved attention, reaction time, and executive attention [94]. Mygind et al. observed improved cognitive performance after waking in natural environments and enhanced self-esteem, mental health, self-efficacy, mood, and concentration following immersive nature experiences, such as camp adventures and outdoor classes [113].

#### Respiratory Health Outcomes

Seven reviews investigating childhood exposure to green spaces and respiratory health outcomes, particularly asthma, exhibited mixed findings in contrast to the consistent positive effects observed for neurodevelopmental and academic outcomes. Lambert et al. found conflicting evidence, with 8 out of 15 studies indicating a protective effect of residential greenness on childhood asthma, two suggesting a detrimental effect, and four reporting no association. [114]. Cao et al. found no significant associations between allergic rhinitis and greenness exposure, although one study indicated a significant increase in the risk of allergic rhinitis [115]. Additionally, Parmes et al. reported no significant association and suggested that nearby green spaces might exacerbate allergic and respiratory symptoms due to potential pollen, mould, and aerosol sources [116]. Hartley et al. found no direct relationships between greenness and child asthma in 6 out of 7 studies, although one study reported a 6% lower risk of asthma associated with greenness, and three papers suggested that greenness was protective of childhood asthma by mediating other adverse effects (e.g. tobacco smoke exposure) [117]. Ye et al. reported inconsistent results, with some studies indicating a higher risk of asthma, childhood wheezing, and allergic rhinitis, while others reported positive effects [102]. Despite heterogeneity and inconsistent results in Mueller et al.’s review, other studies indicated improved lung function in school-age children with higher surrounding levels of greenspace [118]. Finally, Fyfe-Johnson et al. reported positive associations with allergy and asthma in 13 studies, while 8 reported negative ones [103**•**].

#### Cardiovascular Health and Related Risk Factors

Several studies show significant beneficial effects of green space exposure on BP in children [119]. Notably, lower blood pressure levels were observed in children in a forest environment compared to children in non-forest environments [109]. Also, significant beneficial effects and lower odds of hypertension and suggested positive effects on cardiovascular and metabolic measures were observed [102**•**, 103**•**].

Additionally, some studies reported higher levels of PA in areas with more access to green space, such as parks, lower prevalence of childhood obesity, and decreased CVD prevalence [120]. Three reviews added that those who performed PA in parks or other green outdoors would engage for longer moderate to vigorous PA compared to non-green environments, which is positively associated with body weight and health behaviour knowledge [120–122]. Moreover, adolescents who exercised on the beach as a result of living near the beach were more likely to meet the PA guidelines than those living further away [96]. Further, children and adolescents living near the sea reported lower levels of overweight status [96].

## Discussion

This scope review summarizes the epidemiological evidence of short-term and long-term effects of air pollution, noise, heat, and green and blue space exposures on children’s and adolescents’ development and learning performance, particularly on cognitive and mental health, cardiovascular health, and respiratory health outcomes. Ninety-five systematic reviews and meta-analyses were included in this paper, all published in the last six years (2017–2023). Most of the included reviews focused on urban settings, with varying exposure being assessed at home or in and around schools. To our knowledge, this is the first scope review that has synthesized the available evidence regarding the health effects on children and adolescents of air pollution, noise, heat, and green and blue spaces exposure.

### Key Findings

The evidence shows that short- and long-term exposure to traffic-related air pollution, heat, traffic noise, and green and blue spaces can significantly affect children’s and adolescents’ respiratory, cardiovascular, and cognitive health and learning performance. Overall, the most researched environmental exposure was traffic-related air pollution (*n* = 25), specifically, PM_2.5_ (*n* = 26) and NO_2_ (*n* = 10). The evidence reported that PM_2.5_ and PM_1_ were the most damaging due to their very small diameter size, being easily inhaled and penetrating further into pulmonary alveoli, causing stronger adverse effects. Importantly, motorized traffic constitutes the major part of ambient PM_1_ and PM_2.5_ in urban environments, from exhaust emissions and brake wear to road-tire interactions, highlighting the importance of eliminating traffic around school environments [52, 123]. The most researched outcomes associated with air pollution were respiratory health outcomes (see Table 1). Extensive evidence confirms that air pollution increases the risk and prevalence of asthma and asthma-related exacerbations. Further, pollutant exposure can impair cognitive functions and increase behavioural disorders and inattention problems, resulting in lower academic achievements. Furthermore, it is worth noting that in some reviews, low air pollution levels were seen to affect health demonstrating that even in less-polluted countries, necessary measures to reduce air pollution are necessary. Regarding traffic noise, less research exists compared to air pollution, with the studies included in the reviews being of predominantly low quality. Nonetheless, these studies already show some effects on attention, behavioural outcomes, and learning performance. Moreover, heat exposure was the least researched on its effect on health. Yet, respiratory infections seemed to increase, and physical activity levels decrease with hot temperatures. Green space exposure has also been extensively researched (*n* = 25), with significant positive effects observed on cognitive development, attention and behaviour, mental health and wellbeing, learning and academic performance, as well as being beneficial for blood pressure and hypertension and likely increasing physical activity levels and reducing overweight and obesity prevalence. Nonetheless, the reviews assessing respiratory health and green exposure showed contradicting results, in part, because of the high heterogeneity of exposure and outcome assessments. However, the impacts of blue space exposure (*n* = 3) on children’s health require more investigation to confirm the reported health benefits (Table 2).

**Table 2:** Evaluation of the degree of evidence for each exposure and outcome combination

Exposure	Cognitive health	Behavioural outcomes	Learning performance	Mental health	Respiratory health	Cardiovascular health and related risk factors
TRAP	Sufficient	Sufficient	Sufficient	Limited	Sufficient	Limited
Noise	Limited	Limited	Limited	No studies	No studies	No studies
Heat	No studies	No studies	No studies	No studies	Inadequate	Inadequate
Green space	Sufficient	Sufficient	Sufficient	Sufficient	Inadequate	Limited
Blue space	No studies	No studies	No studies	Inadequate	No studies	Inadequate

Sufficient: if most of the studies report an association, but evidence is not yet conclusive enough to confirm that there is a causal relationship; Limited: several good quality and independent studies report an association, but the evidence is still not strong enough; Inadequate: emerging evidence for an association based on some studies; No studies: no studies have been conducted or not enough to conduct a systematic review.

### Limitations and Strengths

Several important limitations need to be considered in the present scope review. First, the cross-sectional design of most of the primary studies prevents us from confirming causality based on the associations observed. Indeed, most reviews have reported the low quality of several studies they have included. Also, the short-term follow-up periods in intervention and longitudinal studies have restricted the availability to evaluate the long-term effects of these exposures or interventions. Thus, the methodological quality of some of the studies included in the reviews needs to be considered. Moreover, meta-analyses, underused in the reviews, may provide more precise effect estimates of the associations than individual studies and address the detection of publication bias. However, a meta-analysis is not an option in systematic reviews with high methodological or statistical heterogeneity and with a limited number of studies, as was the case in most of the included reviews. Moreover, a considerable number of reviews did not report a priori well-developed protocol, which may have increased the risk of sampling bias, selection bias, and within-study bias, as noted in by the amount of “not applicable (N/A)” responses collected on the table in Appendix C (see Supplementary Material). These flaws might have reduced the quality of the synthesized evidence and reporting standardization. In addition, this scope review was not specifically designed to evaluate factors related to soil and water pollution in the urban context, which may have omitted important sources of pollution that can affect children’s health as well. In addition, while the health effects on the prenatal and postnatal phases were not analysed in this review, as these were outside the scope of the present work, evidence shows that urban environmental exposures start affecting health at these early stages of life, and efforts and measures to prevent harmful exposures already from pregnancy should be undertaken [56, 60, 124, 125].

Lastly, our scope review could only synthesize the associations reported in the up-to-date publications of systematic reviews and meta-analyses in the two selected databases. Thus, we might have missed or underestimated associations not included in these systematic reviews. For instance, the latest primary studies of high quality may have been neglected. Nonetheless, this paper also has strengths. First, this is the most comprehensive “tertiary-level” article that synthesizes and assesses the evidence on urban-related exposures (i.e. air pollution, noise, heat, and green and blue space) and children and adolescents’ health using a pooled sample of systematic reviews and meta-analyses. These “tertiary-level” studies are higher on the evidence hierarchy compared to other study designs, including primary and secondary-level studies. Another key strength is that the present review provides additional knowledge concerning both risk and protective factors of urban-related environmental exposure on child health.

### Implications for Policy, Practice, and Future Research

The present review presents evidence that shows how urban exposures can significantly affect the physical and mental development of children and adolescents. Some of these adverse effects can negatively impact their adult life, even beyond health, such as their academic opportunities.

Considering the time children and adolescents spend daily in the school environment, significant changes to reduce exposure to these pollutants can start by transforming school environments. These settings are crucial in providing healthy environments for children and adolescents. Moreover, the location of schools and their neighbourhood characteristics determine different levels of exposure to hazardous and health-promoting elements that can create and widen health inequality. Schools near roads are more likely to reach the WHO air quality guidelines for NO_2_. At the same time, those situated in neighbourhoods with less traffic congestion and more green space are more likely to have better air quality [126]. Reducing traffic around schools and increasing green cover within and outside the school grounds can positively affect their health by reducing their exposure to harmful pollutants. Indeed, as the review has shown, nearby nature and activities in natural spaces offer many benefits for children and adolescents, including stress reduction, increased attention, prosocial behaviour, and increased physical activity.

Moreover, increasing the green and pedestrian spaces in the surrounding area would diminish the proportion of the area used by cars and consequently yield lower pollution levels. Several initiatives are already functioning to change the school environment in different countries. For instance, in the UK, School Streets is an initiative to restrict motorized traffic in and around schools by temporarily closing the street to motorized transport to reduce emissions in these environments and promote active and public modes of transport [13]. Another initiative is *Entornos Escolares Seguros y Saludables* (School environments) based in Spain and created by the Seminario de Movilidad e Infancia, hosted by the *Centro Nacional de Educación Ambiental* (National Centre for Environmental Education). The initiative aims to improve the school environment, reduce harmful exposures, and increase children’s independence by allocating more urban space for active travelling and playing [127]. This initiative promotes changes in the school environments by providing ten measures to effectuate these environments, among other resources found on their website (https://entornosescolares.es). Lastly, another intervention is Protegim les Escoles (Let’s Protect the Schools), a Spanish-based initiative aimed at transforming schools’ surroundings and protecting children from harmful exposures through various actions (e.g. calming traffic measures around schools, pedestrianization of streets and areas, installation of safety elements) [128].

Moreover, children are also highly exposed to TRAP while commuting to school, which often coincides with traffic pollution peaks [129]. Although the time spent during commuting is relatively short (an average of 6% of their daytime), children receive up to 20% of their daily dose of back carbon [38], which is comparable to the 35% of their daily dose received while being at home [129]. Reducing their exposure on their route to school can be achieved at the municipal level, for instance, by introducing low emission zones (LEZs), low-traffic neighbourhoods (LTNs), or pedestrianizing the streets nearby the schools and within the schools’ neighbourhoods. LTNs are areas where vehicle access is restricted to residential streets through barriers and cameras, among others. An evaluation of three LTN schemes in London showed reduced traffic volume and nitrogen dioxide pollution inside the perimeters and on boundary roads [130]. Another strategy is pedestrianization, which eliminates or restricts traffic in a particular street, leaving it for pedestrian use only. For instance, the Spanish city of Pontevedra banned car access in the centre and converted the streets into public spaces for pedestrians to actively commute [131]. The literature shows that to reduce motorized travel and increase active transportation, the built environment needs to be intervened to be adequate for walking and cycling. Active trips require attention to factors deemed less important to motorized travel, such as slopes, safety, security, inclement weather, insufficient lighting, air pollution, and even aesthetics [132]. Specific amenities, such as parks and open spaces, street trees, and urban design qualities, like human scale, transparency, and imageability, are also crucial to active travellers [133]. In their review, Mueller et al. developed ten health-promoting urban and transport principles to ensure that health is integrated into the design of a city’s transport system [118].

#### Future Research

The current evidence presented in this scope review reflects the great impact urban environmental exposures can have on children’s health, advocating for the need to act on mitigating them. Considering that 99% of the world’s population is breathing polluted air [134] and that the population is expected to grow by over 3 billion by 2050 [135], policies to improve the current highly polluted urban environment should be implemented in most countries, especially for the future of children and adolescents, given their vulnerability. However, the evidence is mainly drawn from cross-sectional studies with high heterogeneity levels, of moderate to low quality, and from higher-income countries. Thus, while there is consistent evidence of the associations between these environmental exposures and health, the results cannot represent all urban communities, especially those in lower to middle-income countries. We suggest that future research should focus on better-designed longitudinal and intervention study designs to test for cause and effect, with proper standardized tools to measure the exposures, and include study populations from low- and middle-income countries. Also, studies should include “gold standard” health outcome measures and full adjustment for confounding factors (e.g. socioeconomic background, ethnicity, gender). Moreover, the exact mechanisms underlying the association between environmental exposures and health outcomes are complex and remain unclear and need to be further explored.

## Conclusion

This scope review contributes to the evidence of the health effects of short- and long-term exposure to urban air pollution, leading to a range of harmful effects on children’s and adolescents’ cognitive, respiratory, and cardiovascular health. Children are more susceptible than adults to heat exposure, increasing their risk of its harmful effects on health. Moreover, children’s exposure to traffic noise adversely affects their academic performance and cognitive health. On the contrary, the evidence demonstrates various health benefits of children’s exposure to green and blue spaces, such as stress relief and increased physical activity. Henceforth, the available evidence helps to consolidate the idea that the urban setting in which children live and spend most of their time, such as the school environment, influences their health, clearly stating the need to transform them into health-promoting environments and ensure the healthy development of children and future generations to come. While the review shows that more research is needed to fully understand the mechanisms of how air pollutants affect children’ and adolescents’ health and to conclude the observed associations between heat and noise exposure and children’s health, policy changes and future urban and transport planning interventions that minimize air pollution and traffic noise exposures and increase urban vegetation should be prioritized and put into action to protect this vulnerable lifespan phase to promote healthy adult lives in the future.

### Supplementary Information

Supplementary file 1

## Data Availability

Not applicable.

## Abbreviations

TRAP: Traffic-related air pollution
PM: Particulate matter
UFP: Ultrafine particles of particulate matter
NO2: Nitrogen dioxide
CO: Carbon monoxide
O3: Ozone
PAHs: Polycyclic aromatic hydrocarbons
BC: Black carbon
EC: Elemental carbon
VOCs: Volatile organic compounds
Pb: Lead
Hg: Mercury
Cd: Cadmium
Al: Aluminium
Cu: Copper
Cr: Chromium
Ba: Barium
NDVI: Normalized difference vegetation index
WHO: World Health Organization
UHI: Urban heat island effect
CVD: Cardiovascular disease
BMI: Body mass index
PA: Physical activity
CNS: Central nervous system
ASD: Autism spectrum disorder
ADHD: Attention deficit and hyperactivity disorder
COPD: Chronic obstructive pulmonary disease
AR: Allergic rhinitis
URTI: Upper tract respiratory infection
FEV: Forced expiratory volume
GPA: Grade point average
IQ: Intelligence quotient
OR: Odds ratio
