# Correction: The Health-Related and Learning Performance Effects of Air Pollution and Other Urban-Related Environmental Factors on School-Age Children and Adolescents—A Scoping Review of Systematic Reviews

**DOI:** 10.1007/s40572-024-00448-5

**Published:** 2024-04-17

**Authors:** Inés Valls Roche, Mònica Ubalde-Lopez, Carolyn Daher, Mark Nieuwenhuijsen, Mireia Gascon

**Affiliations:** 1 ISGlobal, Parc de Recerca Biomèdica de Barcelona–PRBB, C/ Doctor Aiguader, 88, 08003 Barcelona, Spain; 2 Universitat Pompeu Fabra (UPF), Barcelona, Spain; 3 CIBER Epidemiología y Salud Pública (CIBERESP), Madrid, Spain

**Correction: Current Environmental Health Reports**

10.1007/s40572-024-00431-0

The originally published version of this article contained mistake, and the author would like to correct them.

Since publication, the authors have become aware of an error in their paper that they are now correcting. Specifically, a citation was missing in the reference list (reference 26) which led to a mismatch between the in-text citations and the references in the reference list from the 26th reference until the last one. Thus, in-text reference 26th is incorrectly corresponding to reference 27th in the reference list, and so on. However, this error does not affect the findings or conclusions of the paper as the content and the articles included remain the same. The authors have generated a new pdf and have added the missing reference and revised that all in-text citations match with their corresponding reference in the reference list.

The original article has been corrected.

