# Supplementary material for: The Health-Related and Learning Performance Effects of Air Pollution and Other Urban-Related Environmental Factors on School-Age Children and Adolescents—A Scoping Review of Systematic Reviews
Source: Curr Environ Health Rep. 2024 Feb 19;11(2):300–16. doi: 10.1007/s40572-024-00431-0 (PMC11082043; doi:10.1007/s40572-024-00431-0)

APPENDIX B

The following search strings with key terms and mesh terms related to the environmental exposures and health outcomes of interest and learning performance outcomes were applied in the search conducted in the 11th of May 2023, for the two electronic databases PubMed and ScienceDirect.

Search string for PubMed:

("Air Pollution"[Title/Abstract]) OR ("Environmental Pollutants"[Mesh] OR "Air Pollutants"[Mesh] OR "Environmental Exposure"[Mesh] OR "Environmental Pollution"[Mesh])) OR "Metals, Heavy"[Mesh] OR "Particulate Matter"[Mesh]) OR PM2.5[Title/Abstract] OR "Fine particulate matter"[Title/Abstract] OR "Extreme Heat"[Mesh] OR "Heat Stroke"[Mesh] OR "Heat Stress Disorders"[Mesh] OR "Heat Exhaustion"[Mesh]) OR (noise) ("Noise"[Mesh] OR "Noise, Transportation"[Mesh] OR "Hearing Loss, Noise-Induced"[Mesh]) OR ("Vehicle Emissions"[Mesh] OR "Traffic-Related Pollution"[Mesh]) OR "Nitrogen Dioxide"[Mesh]) OR ("Ozone"[Mesh] OR "Stratospheric Ozone"[Mesh]) OR "Polycyclic Aromatic Hydrocarbons"[Mesh]) OR "Green spaces"[Title/Abstract] OR "Urban green spaces"[Title/Abstract] OR "Residential greenness" OR "Residential land cover" OR "Temperature"[Title/Abstract] OR "Heat"[Title/Abstract] OR “NDVI”[Title/Abstract] AND (("Cardiovascular System"[Mesh] OR "Cardiovascular Infections"[Mesh] OR "Cardiovascular Diseases"[Mesh] OR "Heart Disease Risk Factors"[Mesh])) OR ("Respiratory Mucosa"[Mesh] OR "Signs and Symptoms, Respiratory"[Mesh]) OR "Respiratory Tract Infections"[Mesh] OR "Respiratory Tract Diseases"[Mesh] OR "Respiratory Hypersensitivity"[Mesh]) OR ("Severe Acute Respiratory Syndrome"[Mesh] OR "Respiratory Insufficiency"[Mesh]) OR "Asthma"[Mesh]) OR ("Lung"[Mesh] OR "Lung Diseases, Obstructive"[Mesh])) OR ("Lung Diseases"[Mesh] OR "Pneumonia"[Mesh]) OR ("Eczema"[Mesh] OR "Dermatitis, Atopic"[Mesh])) OR ("Rhinitis, Allergic"[Mesh] OR "Rhinitis, Allergic, Seasonal"[Mesh]) OR ("Immune System"[Mesh] OR "Immune System Diseases"[Mesh]) OR "Cognitive Dysfunction"[Mesh]) OR

("Academic Success"[Mesh] OR "Academic Performance"[Mesh] OR "Learning Disabilities"[Mesh] OR "Neurodevelopmental Disorders"[Mesh]) OR ("Attention"[Mesh] OR "Attention Deficit and Disruptive Behavior Disorders"[Mesh] OR "Attention Deficit Disorder with Hyperactivity"[Mesh]) OR "Health"[Mesh]) OR ("Health Behavior"[Mesh] OR "Urban Health"[Mesh]) OR "Pediatric Obesity"[Mesh] OR ("Oral Health"[Mesh] OR "Mental Health"[Mesh])) OR "Health Status"[Mesh]) OR ("Environmental Health"[Mesh] OR "Environment and Public Health"[Mesh]) OR ("Infant Health"[Mesh] OR "Adolescent Health"[Mesh] OR "Child Health"[Mesh]) OR ("Depression"[Mesh] OR "Depressive Disorder"[Mesh] OR "Bipolar Disorder"[Mesh])) OR "Exercise"[Mesh] OR "Anxiety"[Mesh]) OR "Anxiety Disorders"[Mesh]) OR "Hypertension"[Mesh]) OR "Blood Pressure"[Mesh]) OR "Child Mortality"[Mesh])) AND (("Child"[Mesh] OR "Child, Preschool"[Mesh])) AND ("Adolescent"[Mesh] OR "Adolescent Development"[Mesh])

Search strings for ScienceDirect:

1. (air pollution) (traffic-related air pollution) (pollutants)(environmental exposure) (heavy metals) traffic AND cognitive neurodevelopment cardiovascular respiratory academic performance obesity diabetes Physical activity attention behaviour AND children OR Adolescents

2. (green space) (green exercise) (green exposure) (blue space) AND cardiovascular respiratory health cognitive neurodevelopment academic performance obesity diabetes Physical activity attention behaviour AND children OR adolescents

3. noise (traffic noise) traffic AND cardiovascular respiratory health cognitive neurodevelopment academic performance obesity diabetes Physical activity attention behaviour AND children OR adolescents AND systematic review OR meta-analysis

4. heat temperature (urban heat island effect) (extreme heat) AND cardiovascular respiratory health cognitive neurodevelopment academic performance obesity diabetes AND children OR adolescents AND systematic review OR meta-analysis

Flow chart – Search May 2023

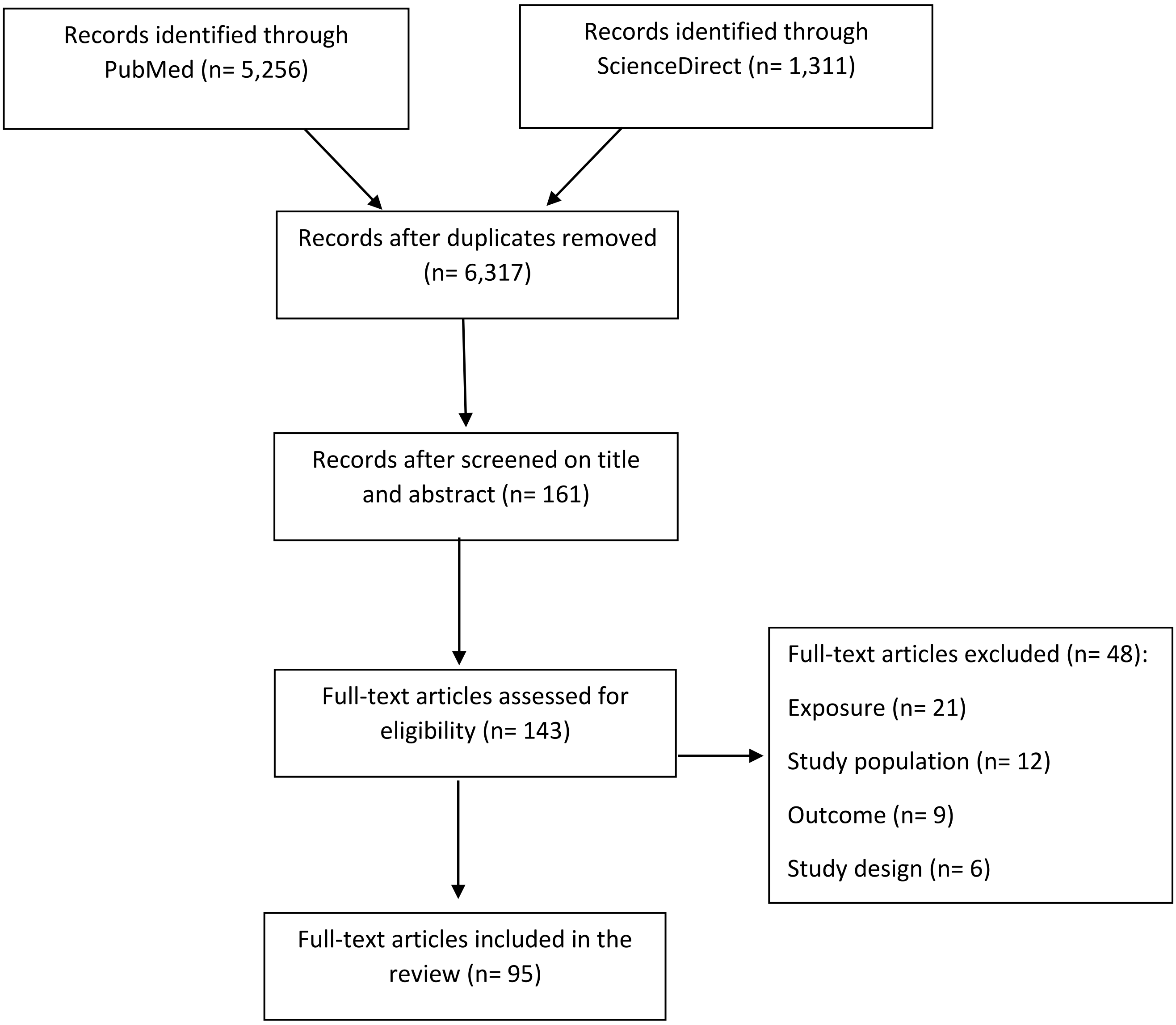

Supplement: Supplementary file 1 [file 40572_2024_431_MOESM1_ESM.zip › Appendix B- search strategy.pdf]
