# Supplementary material for: The Health-Related and Learning Performance Effects of Air Pollution and Other Urban-Related Environmental Factors on School-Age Children and Adolescents—A Scoping Review of Systematic Reviews
Source: Curr Environ Health Rep. 2024 Feb 19;11(2):300–16. doi: 10.1007/s40572-024-00431-0 (PMC11082043; doi:10.1007/s40572-024-00431-0)
Supplement: Supplementary file 1 [file 40572_2024_431_MOESM1_ESM.zip › Appendix C-table of reviews.pdf]

Title	Study (first author & year)	Total number of studies included (N)	Year of publications included	Exposure	Number of studies for each exposure (k)	Health outcome	Study population	Sample size (n)	Geographic location	Method for study quality evaluation	Effect estimates & Effect size	Quality/Heterogeneity	Limitations	Findings
Perinatal and childhood exposure to environmental chemicals and blood pressure in children: a review literature 2007-2017	Sanders, et al. (2018)	32	2007-2017	PM10; PM2.5; Nox or NO2; O3; SO2; CO; BC; PAHs; distance to roadway/traffic/point source; nanosized ultrafine particles; As; Pb; Cd; Hg; phthalates ; Bisphenol A (BPA) ; polychlorinated biphenyls (PCBs); pesticides; flame retardants	10, 8, 8, 5, 3, 2, 2, 1, 3, 1, 2, 2, 4, 3, 4 3, 2, 4, 1	CV health- BP	Age: newborn to 19 years of age	N/A	US, The Netherlands, China, Germany, UK, Saudi Arabia, Pakistan, Belgium, Mexico, Italy, Bangladesh, Irán, Canada, Spain, Korea, Greece, Ecuador	N/A	N/A	The article notes a high heterogeneity of the reported exposure and outcome measures of the studies included	N/A	Among the studies, there were significant associations between air pollution and BP, between PM2.5 and elevated BP, air pollution and BP, all in children. Among the 10 studies of PM10, and 8 studies of PM2.5, 4 and 2 reported significant associations with childhood BP, respectively. Five out of 7 studies of NOx, 4 out of 5 of O3, 2 out of 3 of SO2, 1 out of 2 of CO, and 1 out of 2 of BC reported associations with childhood BP. They found significant associations between Pb, inorganic arsenic, and Cd exposure and childhood BP. None found association with Hg and BP. Lastly, limited evidence suggests exposure to metals during prenatal period may be more detrimental than postnatal exposure
Early life stress, air pollution, inflammation, and disease: an integrative review and immunologic model of social-environmental adversity and lifespan health	Olvera Alvarez, et al. (2018)	16	1998-2018	Air pollution (PM10, PM2.5, NOx or NO2, O3, SO2, CO, BC, PAHs)	16	Immune system- Stress and inflammation	Children, adolescents and adults	Sample ranged from 15 to 2,371,907 individuals	N/A	N/A	N/A	N/A	N/A	Air pollution (PM2.5 and ozone) appeared most detrimental to socially disadvantaged individuals (e.g., racial minorities or low-income households). Although rarely studies evaluate social disadvantage and air pollution affect, air pollutants' negative health effects are closely intertwined with the social stressors, which are also more concentrated among those who are really disadvantaged. Evidence also shows that early life stress and air pollution are likely to have joint effects on health by ways of stress exposure in early life increasing individuals' inflammatory response to particulate air pollution across the lifespan. Many of the diseases (i.e., cardiovascular disease, autoimmune diseases, lung cancer) have an underlying inflammatory component, and air pollution and stress seem to influence the same inflammatory processes (i.e., inducing oxidative stress, activating NF-kB and TLR4.
Cumulative lifetime burden of cardiovascular disease from early exposure to air pollution	Kim, et al. (2020)	34	2019-2022	PM2.5, PM1.0, NO2, TRAP, black carbon	N/A	CV health- BP	Included prenatal exposure, school children, adolescents and adults	N/A	N/A	N/A	N/A	N/A	N/A	Schoolchidlren exposed to UFP or PM2.5 in combination with NO2 demonstrate increase BP. Moreover, PAHs exposure during prenatal and postnatal periods is seen to increase prevalence of overweight and obesity during adolescence. Other studies found significant associations between TRAP and rapid weight gain or higher BMI. Fine PM is known to be highly toxic to cardiopulmonary system. PM2.5 is specially harmful as it penetrates the pulmonary alveoli and induce local inflammatory response. UFP can penetrate the alveolar-capillary barrier and enter the bloodstream. Some of the PM components (PAHs) iniciate monocyte inflammatory responses mediated by reactiveoxygen species and trasition metals.
Exposure to particulate matter: a brief review with a focus on cardiovascular effects, children, and research conducted in Turkey	Elkama, et al. (2021)	N/A	N/A	PM (PM2.5 and PM10)	N/A	Cardiovascular health - blood pressure, inflammation, and hypertension	N/A	N/A	N/A	N/A	N/A	N/A	N/A	Short- and long-term PM and PM2.5 exposure is a major contributor to CV toxicity. Short-term exposure to PM triggers autonomic response, while long-term exposure triggers inflammatory response. Indirect PM mechanisms include lung oxidative stress and inflammation by upregulating pro-inflammatory cytokines and chemokines; also, autonomic imbalance and activation of the sympathetic nervous system. Children are a vulnerable group as PM exposure can start in-utero, and children also have a higher absorption, higher number of alveoli, and more permeable respiratory tract.
Associations between ambient air pollutants and blood pressure among children and adolescents: a systematic review and meta-analysis	Yan, et al. (2021)	15	1996-2017	Pm2.5, PM10, PM1.0, NO2, SO2, O3 (measured per 10 ug/m3 increase)	12, 15, 1, 11, 5, 6	Hypertension and blood pressure (BP)	Schoolchildren and adolescents (from 4y/o to 18 y/o	Sample sizes ranged from 130 to 194,104, and a total of around 0.5 million children and adolescents from China and other HICs	China, Germany, Netherlands, Belgium, and the majority in other countries of Europe and North America	JBI	Increased risk of hypertension associated with long-term PM10 (OR= 1.17, 95%CI: 1.13-1.21); for systolic BP, short-term PM10 exposure was associated (B= 0.26; 95%CI: -000, 0.53), long-term PM2.5 (B= 1.80, 95%CI: 0.94-2.65), and PM10 (B=0.50, 95%CI: 0.10-0.81).For DBP levels, short-term PM10 exposure was associated (B= 0.32, 95%CI: 0.10-0.45).	Not significant heterogeneity between included studies in exception of two for short-term exposure to PM10 and SBP (I2= 96.50%, and I2=82.20%).	No conclusions could be drawn on regards other particles than PM10 and Pm2.5 and hypertension or BP due to limited number of studies.	It was seen an increased risk of hypertension in children per 10 increments of PM10 in long-term effect. Elevated blood pressure was affected by short-term PM10 exposure, long-term PM2.5, PM10, and NO2 per 10 ug/m3 increase. Yet, the results showed to be have more impact long-term exposure to PM2.5 than PM10 on elevated blood pressure. PM2.5 can penetrate into the pulmonary alveoli and enter into the bloodstream, causing to the cv system damage. After sex stratification, risk for increased blood pressure due to PM10 exposure was seen higher in boys.
Effects of ambient air pollution on blood pressure among children and adolescents: a systematic review and meta-analysis	Huang, et al. (2021)	14	1997-2017	PM2.5, PM10, & NO2 and their long-term (>30 days) or short-term (<30 days) exposure	10, 11, 6	Blood pressure on children and adolescents	Children of mean ages of 5.4 to 12.7 years old	A total sample size of 351,766 participants, with	China (8), The Netherlands (n=2), Belgium, Germany, The UK, Europe (UK, France, Spain, Lithuania, Norway and Greece)	NOS	Meta-analysis for short-term exposure to air pollution showed only exposure to PM10 was significantly associated with SBP values. Meta-analysis for long-term exposure to air pollution showed to be generally significant in cross-sectional studies.	Significant heterogeneity for association between long-term PM10 exposure and SBP/DBP. (Heterogeneity I2 over 50% means that it is significantly high) For long-term effects, meta-analysis of PM10 and SBP (91.50%), NO2 (54.82%) and PM10 (90.65%) with DBP. For short-term effects, PM10 (95.99%) with SBP, PM2.5 (70.6%) and DBP, and PM10 (97.19%) and DBP.	No meta-analysis could be conducted for O3, NOx, SO2, and CO due to limited number of studies.	Short-term exposure to PM10 was significantly associated with elevated SBP values. Long-term exposure to PM2.5, PM10, and NO2 is significantly associated with increased BP among children and adolescents. The exact mechanisms by which these exposures contribute to elevated BP remain unknown for the authors.

Long-term association of ambient air pollution and hypertension in adults and in children: a systematic review and meta-analysis	Qin, et al. (2021)	57 studies, but only 4 studies on children	1980-2018	PM1, PM2.5, PM10, NO2, NOx, SO2, and O3 (measured per 10 ug/m3 increase)	1, 2, 2, 1, 2, 2 (in children population)	Hypertension (a systolic blood pressure)	Samples of children and adults	that ranged from 587 to 39,348,119 participants among the studies included	China (all children's studies)	NOS	The summary ORs (95% CI) per 10 ug/m3 increase in PM2.5, PM10, SO2, and O3 in children were 2.82 (95%CI: 0.51-15.68), 1.15 (95%CI: 1.01-1.32), 8.57 (95%CI: 0.13-575.58), and 1.26 (95%CI: 0.81-1.09).	Median quality score was 8 for cohort studies, 6 for case-control studies, and 7 for cross-sectional. Overall, the 41 studies were rated as high quality. Heterogeneity (I2) for Pm2.5, PM10, SO2, and O3 was 83.8%, 0, 94.2%, and 91.6%. Thus, high heterogeneity for PM1, PM2.5, NOx, SO2, and O3, moderate heterogeneity for NO2, and small for PM10.	The limited number of studies based on children and all based in China can be a limitation for interpreting the results as conclusive.	All the studies (n=4) conducted on children and not adults were based in China. They found significant increase in hypertension or significant increase in risk of hypertension for PM1, PM2.5, PM10, NO2, SO2, O3, and CO exposure.
Association of ambient air pollution with blood pressure in adolescence: a systematic review and meta-analysis	Tandon et al. (2023)	8		PM10, PM2.5, NO2, O3, SO2, CO, PM1	5, 6, 7, 3, 1, 1, 1	BP	10 to 18 yr	100 to 9345 - a total of about 15,000	Europe (n=5), China (n=2), USA (n=1)	GRADE	β values and 95%CI. Significant associations for NO2 and DBP (β= 5.56 (1.23, 9.99) mmHg).	Overall quality was low and very low.	Very few studies of very low quality, with high heterogeneity between geographic locations	Only in the cross-sectional studies they observed significant positive associations between long-term exposure to PM10 on SBP and DBP, and for NO2 on DBP only. In a another subgroup analysis of 12 year-old adolescents, they found significant positive associations for long-term exposure to PM2.5 and PM10 on DBP. For results that could not be pooled, they observed significant positive associations for long-term PM2.5 on SBP, and O3 on SBP and DBP.
Early environmental exposures and life-long risk of chronic non-respiratory disease	Vilcins, et al. (2021)	N/A	N/A	Environmental exposures (toxic metals, PM)	N/A	Noncommunicable diseases - Cardiovascular disease, neurodevelop and neurodegeneration	N/A	N/A	N/A	N/A	N/A	N/A	N/A	Prenatal exposure to phthalates and perfluorinated compounds is associated with LBW and preterm birth, which these are associated with type 2 diabetes. Many studies found a synergy effect of indoor and outdoor air pollution exposures on the development of diabetes type 2 and obesity. Also, children exposed to PM have rapid postnatal weight gain and reach higher body mass index. Lead is the most well-known to affect cognitive health of children and behavioural disorders. Air pollution exposure at home or school has been associated with increased risk of ADHD. Mercury exposure is associated with increased odds of ASD and ADHD.
The association between childhood exposure to ambient air pollution and obesity: a systematic review and meta-analysis	Huang, et al. (2022)	15	1998-2019	PM10, PM2.5, PM1, O3, and NO2	9, 11, 3, 2, 11	Childhood obesity	Ages ranged from 2 to 18 y/o	Total sample size of 683,081 children and adolescents	China (n=6), Spain (n=3), USA (n=2), Italy, Mexico, Netherlands, & UK	The National Institute of Health's Quality Assessment Tool for Observational Cohort and Cross-sectional Studies for study quality assessment (score 0-14).	Meta-analysis showed that long-term exposure to O3 had no significant correlation with risk of childhood obesity (OR=1.08); while the association for PM10, Pm2.5, PM1, and NO2 was (OR= 1.12, 95%CI: 1.06-1.18), (OR=1.28, 95%CI: 1.13-1.45), (OR= 1.41, 95%CI: 1.30-1.53) and (OR= 1.11, 95%CI: 1.06-1.18).	Quality assessment showed that 8 studies scored a 13, while the others 11 or 12. Thus rated as of good quality. Heterogeneity was found in studies with obesity as outcome for PM10 (85.9%), PM2.5 (86.3%), O3 (71.5%), NO2 (84.1%), except for PM1 (0%). For the outcome of BMI status, heterogeneity was found among all pollutants, PM10 (89.1%), PM2.5 (82.6%), NO2, 48.6%), and NOx (91%).	N/A	Significant association between PM and NO2 and risk of childhood obesity, and increased BMI. Although O3 and NOx had positive effects in the increase in weight status, noen reached significant levels. Notably, as the aerodynamic of the PM decreases, the fattening effects on children increases, which is consistent with previous studies' hypothesis.
Exposure to outdoor and indoor air pollution and risk of overweight and obesity across different life periods: a review	Shi et al. (2022)	18	2012-2021	Outdoor PM2.5, NO2, TRAP, PM10	N/A	Obesity - BMI	0 to 17 yr	565 - 40,953	China (n=3), USA (n=6), Spain (n=1), Netherlands (n=1), Europe (n=1),	N/A	N/A	N/A	No method for evaluation of evidence and no methodology explicited	In a study, school-age children had a 10% increased risk of obesity per 10 ug/m3 increase in PM2.5 exposure. School exposure to UFP, PM2.5, EC and NO2 was positively associated iwth overweight or obesity in school-age children compared to exposure a home (n=1). And children exposed to the highest quartile of exposure had a higher risk of becoming overweight or obese. Moreover, outdoor PM2.5 absorptive exposure and NO2 in childhood was related to a greater risk of becoming overweight or obese (n=2). In another study, TRAP exposure was certainly linked to increased BMI, and the annual increase in BMI was approximately 13.6%. Increases of 10 ug/m3 in Pm2.5, O3, and NO2 exposure were related to an increased risk of obesity.
Are the adverse effects of air pollution modified among active children and adolescents? A review of the literature	DeFlorio-Barker et al. (2022)	10	2002-2021	Pm2.5, SO2, NO2, O3	N/A	PA (modiable factor), metabolic outcomes (n=4), asthma (n=1), executive function (n=1), and others (n=4)	Children	N/A	China (n=5), USA (n=3), Indonesia (n=1), Germany (n=1)		N/A		This review includes different measurement of PA, also, the intensity of PA might not be taken into account and could influence how AP influences health during PA. Moreover, the review include very few studies with a wide range of health outcomes which make it difficult to compare and contrast.	Five studies noted no increased benefits (n=3), and some evidence of detriments (n=2), from increased PA in higher polluted areas. Other articles suggested that the benefits of PA were diminshed on health in areas of higher pollutant ocncentrations (n=3), two articles found that in areas with high pollution, PA had no positive effect on cardiopulmonary fitness. Two studies indicated a detrimental health efect with increased PA on glucose resistance and asthma development.

Effects of long-term exposure to traffic-related air pollution on lung function in children	Schultz, E. S., Litonjua, A. A., & Melén, EN., (2017)	32	N/A	Traffic-related air pollution (PM, O3, NOx, CO)	32	Lung function (FEV, FEV1, among other measures)	Ages ranged from 5 to 24 years of age.	N/A	UK, China, Canada, Netherlands (n=7), USA (n=7), Europe (n=3), Germany(n= 4), Taiwan, Spain, Sweden (n=3), Norway, Austria, México	N/A	N/A	N/A	N/A	Despite advances with peripheral airway assessment, it is rarely investigated whether TRAp exposure affects small airway infection. Nonetheless, associations between exposure and small airway infections have been reported. Differences in sex are inconsistent among studies, yet, 6 studies showed stronger exposure effect in males compared to females. In regards to whether asthma is an effect modifier for the association TRAP-lung function, almost studies found no effect modification by asthma.
Current knowledge of environmental exposure in children during the sensitive developmental periods	Perlroth, et al. (2017)	N/A	N/A	Environmental exposures	N/A	Respiratory outcomes	N/A	N/A	N/A	N/A	N/A	N/A	N/A	A child breathes more air than an adult at rest, even though the lung capacity of the adult is greater. An infant has three times the ventilation per minute than an adult, and a 6-years-old has twice this volume. Thus, inhaling toxic substance may compromise their pulmonary function. Chemicals (PCBs, methylmercury, lead, manganese, etc.,) may interfere with critical developmental processes of the brain, leading to developmental neurotoxicity through ingestion of contaminated food, and gas inhalation, or skin contact.
Short-term association between ambient air pollution and pneumonia in children: a systematic review and meta-analysis of time-series and case-crossover studies	Nhung et al. (2017)	17	1999-2016	PM10, NO2, O3, PM2.5, and SO2	N/A	Pneumonia - emergency visits, hospital admissions	0 to 18 yr	339 - 90,063	USA (n=6), Brazil (n=6), Chile (n=1), Australia (n=1), Italy (n=1), Spain (n=1), China (n=1), New Zealand (n=1)	No quality assessment	Estimated risks (ER) and relative risks (RR) per 10 ug/m3 increase. The estimated risks per 10 ug/m3 increase for PM10 was 1.5% (95%CI: 0.6%-2.4%), for PM2.5 was 1.8% (95%CI: 0.5% - 3.1%), for O3 was 1.7% (95%CI: 0.5% - 2.8%), for NO2 was 1.4%(95%CI: 0.4%-2.4%)	The degree of heterogeneity in the meta-analysis was large for PM10 (66.1%), NO2 (71.1%), O3 (75.02%) and CO (68.1%)	These results from time-series analyses, which only capture the most immediate acute effects of a few days of exposure instead of capturing the cumulative long-term effects, and thus, the % might be lower	With the exception of CO, all pollutants asssessed were consistently associated with pediatric pneumonia hospitalization. They estimated tht the short-term effect of PM2.5 was around 1.8% (95%CI: 0.5%-3.1%) on pneumonia hospitalization for a 10 ug/m3 increment of PM2.5. These effects were observed in regions were the levels of air pollution are quite low, such as Australia and New Zealand (with daily means of PM2.5 of <11 ug/m3). Larger health risks due to air pollution are observed in low-income countries (e.g., Brazil), compared to high-income countries, such as USA.
Association of outdoor air pollution with the prevalence of asthma in children of Latin America and the Caribbean: A systematic review and meta-analysis	Orellano et al. (2017)	20, 16 for meta-analysis	2004-2015	Pm10, NO2, So2, and O3	N/A	Asthma	N/A	48,442 children	Brazil (n=7), Colombia (n=6), Peru (n=2), Bolivia (n=1), Chile (n=1), Argentina (n=1), French West Indies (n=1), Honduras and El Salvador (n=1)	RoB with NOS	OR and crude Ors with 95%CI. They found a positive association between asthma and outdoor AP: a higher prevalence of asthma in children with living in a polluted environment (OR=1.34; 95%CI: 1.17-1.54).	RoB was low in 6 studies, intermediate in 12 studies, and high in 2 studies. Heterogeneity between studies in the quantitative analysis was moderate (I2: 68.39%)	The exposures of AP included were based on road traffic but also industry and other non-human activites, and no differentiation was made in the results section as to where did the source of pollution come from.	Most studies found a significant positive association between asthma prevalence and at least, one air pollutant (n=14), or between asthma and proximity to a known source of air pollution, while another found a significant negative association (n=1).
Exposure to traffic-related air pollution and risk of development of childhood asthma: a systematic review and meta-analysis	Khreis, et al. (2017)	41	1999-2016	TRAP (measured mostly by using land-use regression models): NO2, PM2.5, BC, PM10, NOx, EC, CO, PMcoarse, NO	31, 18, 15, 14, 6, 4, 3, 3, 2	Respiratory outcomes-asthma	Age ranged from 1 to 21 y/o	Sample sizes ranged from 184 to 1,133,938 participants	Europe (n=17), North America (n=11), Japan (n=5), China (n=3), Taiwan (1).	Critical Appraisal Skills Programme to evaluate study's quality. Standardized concentrations: 0.5 x 10-5 m-1 BC; 4ug/m3 NO2; 30ug/m3 NOx; 1ug/m3 2.5; and 2ug/m3 PM10	Significant increase in asthma development associated with BC exposure (OR= 1.08, 95%CI: 1.03-1.14). For NO2 and PM2.5, there was a statistically significant increase in asthma's development (OR= 1.05, 95%CI: 1.02-1.07; and OR=1.03, 95%CI: 1.01-1.055, respectively). PM10 exposure statistically significantly increase asthma's development (OR= 1.05, 95%CI: 1.02-1.08).	Overall, the selected studies are of a good quality.	Some of the limitations identified were non-representative samples, evaluating asthma by questionnaires, and not adjusting for important cofounders.	Overall, it showed positive and statistically significant associations with four pollutants examined, these pollutants are highly correlated in traffic exhaust. The evidence for BC was less heterogeneous than for PM2.5, and PM10, and in particular for NO2, which may give further indication of any putative agent. The question whether the increase in asthma incidence estimated represents added cases or merely an acceleration of the development of asthma or increased severity making the disease sufficiently apparent for clinical diagnosis is unresolved.
Assessing the impact of air pollution on childhood asthma morbidity: how, when, and what to do	Burbank, & Peden, (2018)	12	N/A	Air pollution	12	Asthma and lung function	Ages ranged from 0 to 18 y/o, and in two studies was from 0/5 to 66/80 y/o	N/A	US	N/A	N/A	N/A	N/A	Exposure to air pollution is linked with poor asthma outcomes, with various studies demonstrating reduced lung function in children, and increased rates of rescued medication use, emergency department visits, and hospitalizations for asthma exacerbation. TRAP prenatal and postnatal exposure has been linked with decreased lung development, and increase in the prevalence of asthma and allergic disease. NO2 is associated with development of atopy, current wheezing, and lower forced respiratory volume in 1 second (FEV1). PM10 exposure during first year of life is associated with reduction in FEV1, and PM10 near home with increase risk of asthma-related hospitalization. Long-term PM2.5 exposure is associated with severe asthma exacerbations, and positively correlated with wheezing episodes in children of 2-10 years old. Both short- and long-term exposure to O3 is associated with negative pulmonary health effects (e.g., lower FEV1, increased asthma-related ED visits).
School exposure and asthma	Esty, & Phipatanakul, (2018)	8	N/A	School environmental exposure	N/A	Asthma morbidity	Population aged from 4 to 15 years of age	Sample ranged from 248 to 6,346	Netherlands, US, Denmark, Taiwan, & Sweden	N/A	N/A	N/A	N/A	In the school setting, pollutant exposures include PM, NO2, BC, O3, bioaerosols, volatile organic compounds, CO. Traffic pollution is also an important source of exposure in the schools as many are in close proximity to a major roadway. Few studies have evaluated the association of school based pollutant exposure and asthma morbidity. Studies have demonstrated that NO2 and O3 were associated with airflow obstruction in children with asthma, and a significant decrease in peak expiratory flow and FEV1 in children with asthma, respectively. Another study found that having more than one classroom trigger (pollutant) increased the likelihood of worsened asthma morbidity in 5-11 year-old children.

Outdoor air pollution and the burden of childhood asthma across Europe	Khreis, et al. (2019)	N/A	2010-2012	Air pollution (NO2, PM2.5, BC)	N/A	Asthma development	Children aged from 1 to 14 years of age	63,442,419 children included	18 European countries; Austria, Belgium, Denmark, Finland, France, Germany, Greece, Hungary, Ireland, Italy, Lithuania, Netherlands, Norway, Portugal, Spain, Sweden, Switzerland, Uk	N/A	N/A	N/A	N/A	It is estimated that 33% of all childhood asthma cases could be attributed to air pollution. The attributable percentage of cases was not sensitive to the use of age-specific asthma incidence rates as shown in the stratified analysis for the age groups 1-4 and 5-14 years old. It was estimated that compliance with the NO2 WHO air quality guideline value and the PM2.5 WHO air quality guideline value could prevent 2434 and 66567 new cases of asthma childhood per year, respectively. Moreover, meeting the minimum levels of NO2, PM2.5, and BC could prevent, respectively, 135257 (23% of all), 191883 (33% of all), 89181 (15% of all) new childhood asthma cases per year. Also found that the attributable percentage of cases varied depending on the pollutant studied (23% for NO2 and 15% for BC) and the exposure reduction scenario investigated.
Impact of ambient air pollution and wheeze-associated disorders in children in Southeast Asia: a systematic review and meta-analysis	Luong et al. (2019)	12, 10 for meta-analysis	1999- 2018	PM110, PM2.5, SO2, NO2, CO, O3	8, 3, 4, 5, 3, 4	Hospital admissions for heeze-associated disorders (n=4), incidence/prevalence of wheeze-associated disorders (n=4) and emergency room visits or mrotlity (n=4)	From <5 to 18 yr	N/A	Thailand (n=4), Vietnam (n=3), Singapore (n=1), Indonesia (n=1), Malaysia (n=1)	Biomed Central for study assessment	Per 10 ug/m3 increase of pollutants: PM10 was 1.020 (95%CI: 0.999-1.041);for PM2.5 1.010 (95%CI: 1.001 - 1.020); for SO2 1.008 (95%CI: 0.998-1.018); for NO2 1.013 (95%Ci: 0.997 - 1.029), for Nox 0.999 (95%CI: 0.999-1002).	Degree of heterogeneity in the meta-analysis was: for PM10 (99.6%), CO (85%), NO2 (94.2%), PM2.5 (57.5%), SO2 (61.7%), Nox (56.3%), O3 (49.6%)	7 out of 10 studies were performed in low-income countries, where children may be exposed to other sources of pollution such as biomass burning at home, tobacco exposure, which could be cofounding bias. Also, very few studies performed on several countries.	The studies confirmed that fine particulate matter (PM2.5, PM1) were signfiicantly associated with wheeze-associated disorders in children. Their meta-analysis showed that PM1 was most strongly associated with wheeze-associated disorders. In regards PM10, the association was not clear. For SO2, they did not observe a significant association. Lastly, for CO, O3, their effects on wheeze-associated disorders has not been proven.
Associations between air pollution and pediatric eczema, rhinoconjunctivitis and asthma: a meta-analysis of European birth cohorts.	Fuertes, et al. (2020)	A meta-analysis of five birth cohorts, not single primary studies	2003-2011	Long-term annual concentrations of NO2, NOx, PM10, PMcoarse, PM2.5	For all cohorts	Respiratory outcomes- eczema, rhinoconjunctivitis, and current asthma	Outcome data from birth cohorts at 4 and/or 8 y/o	N/A	Europe	N/A	The meta-analysis results for eczema, rhinoconjunctivitis, and asthma at 4 years were OR= 0.94 (95%CI: 0.81-1,09), OR= =.90 (95%CI: 0.75-1.09), OR= 0.91 (95%CI: 0.74-1.11), respectively, for NO2 exposure; and OR= 1.00 (95%CI: 0.81-1.23), OR= 0.70 (95%CI: 0.49-1.00), OR= 0.88 (95%CI: 0.54-1.45), respectively, for PM2.5 exposure.	Heterogeneity was moderate (30-50%) between cohort-specific effect estimates for NO2 estimated to the birth and current addresses and the heterogeneity were more marked for PM2.5 (>50%). Heterogeneity was also observed for rhinoconjunctivitis and asthma at eight years for almost all pollutants estimates (50-90%).	N/A	They found no evidence to support the hypothesis that long-term air pollution levels at home address were associated with an increased prevalence of pediatric eczema, rhinoconjunctivitis, or asthma. It should be noted that although a high percentage of European populations are exposed to levels of air pollution that exceed the WHO reference levels, air pollution levels in general have decreased during the last decade. Therefore, these birth cohorts were exposed to lower levels of air pollution compared to other cohorts.
Climate change and childhood respiratory health: a call for action for paediatricians	Di Cicco, et al. (2020)	N/A	N/A	Air pollution and heat and immune and respiratory outcomes	N/A	Respiratory and allergic diseases	Ages ranged from 6 months to 8 years of age	N/A	N/A	N/A	N/A	N/A	N/A	The interpaly amongst CC, air pollution, and allergenic pollens may lead to damage of the airway mucosa through impaired mucociliary clearance, increased permeability and stimulation of the immune response with increased production of IgE. Overall, the results indicate that the temperature may contribute to the global increased prevalence of rhinitis symptoms, altering the timing, spatial distribution, quality and quantity of allergenic plants and pollens. Studies have found that increased temperatures are likely to increase asthma-related emegency visits in children, especially when the exposure to hot temperature is for several days.
Allergic rhinitis aggravated by air pollutants in Latin America: a systematic review	Filho, et al. (2021)	22	1999-2020	Environmental pollutants: PM10, NO2, O3, PM2.5, BC	6, 3, 3, 2, 1	Respiratory outcomes- allergic rhinitis	Ages ranged from 4 to 59 years old	Total sample size of 33,873 children and adolescents (and only 4.94% of adults).	Brazil, Colombia, Bolivia, Argentina, Chile, Perú, Costa Rica.	NOS	The OR for an exposed person to air pollution of experiencing allergic rhinitis was 1.43 (95%CI: 1.026-1980). For children and adolescents the OR was 1.36 (95%CI: 1.051-1759).	The I2 statistic (heterogeneity) was 89.3% and reinforces the hypothesis of heterogeneity. 9 cross-sectional studies had low risk of bias, 8 presented a moderate risk, 1 had a high risk of bias. None of the longitudinal studies had high risk of bias, most had low in exception of one that had moderate risk.	Due to high heterogeneity the result should be interpreted with caution and these are not conclusive but rather suggestive.	It provides evidence that air pollution is a risk factor for allergic rhinitis in Latin American countries, supporting what has been demonstrated in other regions. For children and adolescents, the probability of a person exposed to air pollutants to experience AR is 36% greater. Peculiarities that significantly affect the children are due to biological immunity, prenatal and postnatal lung development, higher energy, and metabolic consumption.
Short-term exposure to ozone, nitrogen dioxide, and sulphur dioxide, and emergency department visits and hospital admissions due to asthma: a systematic review and meta-analysis	Zheng, et al. (2021)	67	1990-2010	Air pollutants: NO2, O3, and SO2 (measures per 8h daily concentrations or average 24h concentrations)	N/A	Asthma exacerbations	Children, adults and elderly	N/A	Most were in developed studies (Europe, and North America), few on LMICs (Latin America and Asia). However, none on Africa.	The quality study was measured using an adaptation of the Grading of Recommendations Assessment, Development and Evaluation approach.	Effects sizes as RRs. The effect size of meta-analysis (and heterogeneity) for long-term exposure for O3 was 1.008 (PI= 1.001-1.015), for short-term exposure was 1.017 (PI= 0.962-1.076), for long-term NO2 exposure was 1.014 (PI= 1.000-1.028), for short-term exposure was 0.999 (PI= 0.961-1.038), for long-term exposure of SO2 was 1.010 (PI=0.987-1.033), for short-term exposure was 1.003 (PI= 0.993-1.013).	The quality of studies investigating long- and short-term (8h) O3 and long-term (24h) NO2 and asthma was high. The level of quality evidence for long-term (24h) SO2 was moderate. The evidence was moderate for short-term O3 and SO2 exposure, and low for NO2.	Heterogeneity between studies in methodology, definitions, and geographical locations may have led to some findings being inconclusive, thus, not a lot of information to report apart from the one written in the main conclusions section.	Higher levels of average 24-hour concentrations of NO2 and SO2, and higher levels of maximal 8-hour or average 24-hour concentrations of O3 were associated with significantly increased risks of asthma-related exacerbations, defined as ERVs. On the contrary, the results of maximal 1-hour daily concentrations for the three pollutants did not show significant association with asthma exacerbations. It could be due to the small number of studies that evaluated this association. The analyses demonstrate that children and, to a lesser extent, the elderly were more susceptible to the adverse effects of air pollution, which was consistent across the three pollutants.

Air pollution and asthma: mechanisms of harm and considerations for clinical interventions	Pfeffer, et al. (2021)	N/A	N/A	Air pollution	N/A	Respiratory outcomes via immunity-related changes	N/A	N/A	N/A	N/A	N/A	N/A	N/A	Although development of allergic sensitization is a vital part of asthma's pathogenesis in most patients, respiratory tract infections cause the majority of acute severe exarcerbations. And air pollution has multiple actions to increase susceptibility to and harm from respiratory tract infections. For example, NO2 exposure increases epithelial expression of ICAM-1, which, among other actions, is the receptor for rhinoviruses. Thus, the mechanism acting in here is impaired mucosal barrier function.
Air pollution and lung function in children	García, et al. (2021)	8	N/A	Air pollution	N/A	Lung function (FEV, FEV1, among other measures)	Children and adolescents	N/A	North America, Europe & Asia	N/A	N/A	N/A	N/A	Short-term exposure to air pollution can reduce children's lung function. Short-term exposure to PM and Ozone is linked with poorer lung function, lower FEV1. Short-term exposure to NO2 levels has been associated with reduction in children's lung function. Studies from Sub-Saharan Africa, Bangladesh, and China show that acute exposure to NO2 and PM2.5 impacts lung function, and cause slower lung function growth. For PM10, the 2 studies did not find any evidence. Ozone's effect on lung function has been less consistent.
Association between ambient air pollution and childhood respiratory diseases in low- and middle-income Asian countries: a systematic review	Ibrahim, et al. (2021)	41	2015-2019	Air pollution (short-term and long-term exposure): PM10, PM2.5, CO, O3, NO2,	N/A	Respiratory outcomes	Children and adults	Sample sizes ranged from 104 to 6,727,439 children. Adults were also included in 9 studies	Malaysia, Iran, Thailand, Turkey, China, Iran, Mongolia, Vietnam, Lebanon, Indonesia, (but most on china)	Study quality assessment was assessed by the NOS (score 0-9), the Mustafic criteria (score 0-5), and the Appraisal Tool for Cross Sectional Studies (ATCS) (score 0-15 points).	N/A	Overall, most of the studies were sufficiently high quality for evaluating the relationship between air pollution exposure and childhood respiratory disease. None were of low quality. 3 out of 5 by the NOS score scored 6, the remaining 5. 22 other studies scored a maximum of 5 by the Mustafic criteria. 5 studies scored a minimum of 17 points with ATCS. None the studies had high risk bias.	The exposure assessments were at district, city or state level. Also, the contribution of other health determinants (e.g., socioeconomic factors, prenatal condition) were not considered in the interpretation of the results.	Ambient air pollution (PM2.5, PM10, SO2, NO2) is associated with acute and chronic respiratory diseases, hospital admissions, patient visits and emergency visits related to respiratory diseases. It has also been linked with asthma occurrence. Inconsistent evidence for CO and asthma-related hospital admissions. Prenatal exposure is linked with wheezing episodes, and postnatal exposure to PM10 was associated with higher remission of wheeze and allergic rhinitis.
A review of the traffic-related air pollution around schools on student health and its mitigation	An, et al. (2021)	127	N/A	TRAP (measured mostly by using land-use regression models): NO2, PM2.5, BC, PM10, NOx, EC, CO, NO	N/A	Cognitive health and respiratory health	School-aged children	N/A	N/A	Conducted meta-analysis with a 95% CI. No tool was used to assess the study quality	A significant increase in overall risk of PM2.5 for respiratory diseases (OR= 1.11, 95% CI= 1.01-1.22). For NO2 exposure, it was not found statistically significant to be associated with asthma, however, it was a risk factor for respiratory disease (OR=1.05, 95%CO= 1.02-1.08).	Statistical significant heterogeneity was detected for PM2.5 and NO2 exposure and risk for respiratory disease (P=0.000; P=0.000).	N/A	Children exposed to low levels of NO2 have an increased reaction time of 14.8s than children exposed to higher concentrations. Exposure to PM2.5 and BC during commuting is associated with reduced growth in working memory. Air pollution exposure has been associated with bronchopneumonia, bronchitis, and acute tonsillitis. Children's pulmonary function is significantly lower at schools with high concentrations. TRAP is linked to increased blood pressure, and long-term exposure can increase the risk of cardiovascular diseases and increased mortality. TRAP exposure can lead to increased allergic symptoms, and atopic allergies. PM exposure is linked to lower GPA.
Interactive effects of allergens and air pollution on respiratory health: a systematic review	Lam,et al. (2021)	35	1994-2019	Airborne allergens (most measured by defining daily allergen exposure): total pollen, total fungal, specific pollens, and fungal types	12,13,22,13	Respiratory outcomes-asthma and allergies	Samples included children, adolescents and adults	N/A	USA (n=12), Australia (n=6), Japan (n=4), Canada (n=3), UK (n=3).	Study quality through using a modified version of a validated study quality instrument.	N/A	Most studies were of good quality. Multiple testing and reporting bias are likely as multiple lags and several allergen-pollutant combinations were frequently tested but interactions effect estimates or p-values reported for only a subset of these analyses.	N/A	Overall, 12 studies have found a significant statistical allergen-pollutant interaction. 8 interactions were related to asthma outcomes, three to medical consultations for pollinosis and one to allergic symptoms. As the relative majority (six) of allergen-pollutant interactions were identified for daily number of asthma hospital admissions or visits, this may indicate that such interactions primarily influence severe asthma symptoms requiring hospitalization. Nonetheless, overall the epidemiological evidence for allergen-pollution interactions found in this systematic review was relatively weak and inconsistent. Only the interactions between Japanese pollen counts and measures of high desert dust levels or PM were reported by more than one study.
Effect of particulate matter exposure on the prevalence of allergic rhinitis in children: a systematic review and meta-analysis	Lin,et al., (2021)	21	N/A	Air pollution: PM10, PM2.5	15, 9	Respiratory outcomes-allergic rhinitis	N/A	Samples ranged from 995 to 39,993 participants with a total of 217,396 among all studies	Asia (n=14), Europe (n=5), and America (n=2)	Meta-analysis results were reported as OR values. Heterogeneity was tested using the Cochran Q statistic test.	Statistical relation between concentration of PM2.5 and prevalence of childhood AR has been found (OR PM2.5= 1.089; 95%CI= 1.010-1.174, p=0.027).Association between PM10 and childhood AR are significant as well (OR=1.063, 95%CI= 1.018-1109, p=0.005). Based on developed/developing countries analysis, the 3 articles in developing countries had a significant statistical difference (OR= 1.165, 95%CI: 1.093-1.241), while the ones in developed countries did not.	Heterogeneity in the studies of association between PM2.5 and childhood AR was I2= 74.1%, p=0.000. Heterogeneity for PM10 and childhood AR association was I2=83.7%, p=0.0033.	N/A	This meta-analysis found that childhood exposure to PM significantly all increased the risk of suffering from AR, the PM being studies mainly was PM2.5, and PM10. A study reported that particulate pollution from vehicles can mediate allergic inflammation, enhance IgE response, and improve airway hypersensitivity. Moreover, it was found that the OR of PM2.5 was larger than for PM10, meaning that PM2.5 exposure was more dangerous than PM10. While the mechanisms of AR cause by PM are not clear, animal studies have found that PM can increase IgE, and can inhibit Th1 lymphocytes, reduce IL-2 activity, and prolonged exposure can cause IgE to increase. Moreover, the meta-analysis found that PM effect on childhood AR was more significant in Asia; yet, most studies from Asia were based in China, which has become increasingly industrialised. Thus, it does not represent the continent. Also, Europe area had an enhancement between PM10 and childhood AR. But not in America. Another difference between subgroups was that children in developed countries were more affected by PM10, while children in developing countries were more affected by PM2.5.

Impact of early life exposure on respiratory disease	Bush, A., (2021)	7	N/A	Air pollution	N/A	COPD and asthma prevalence	Age ranged from 3 to 65 years of age	27,000 participants	N/A	N/A	N/A	N/A	N/A	Early sensitization to multiple airborne allergens is linked to acute severe attacks of wheeze, and impaired growth in spirometry. The evidence showed that lung function will track from preschool years until the sixth decade of life for most people. The groups with highest risk for COPD all associate with childhood adverse factors, with severe childhood asthma being the strongest predictor of COPD.
Impact of environmental justice on children's health-interaction between air pollution and socioeconomic status	Mathiarasan, et al. (2021)	N/A	N/A	Air pollution	N/A	Respiratory and cognitive outcomes	N/A	N/A	N/A	N/A	N/A	N/A	N/a	Children of lower socioeconomic status are exposed to higher concentrations of both outdoor and indoor air pollution. It is likely that air pollution and stress trigger the same biological pathways, and stress may be caused due to being in a low socioeconomic status. Air pollution can also cause neuroinflammation, which is linked to neurodegenerative diseases. And as the growth process is still developing during childhood, changes to their DNA caused by air pollutants can have detrimental health effects, such as inflammation and disease. Moreover, even if children become less exposed to air pollution as young adults, they are still more likely to have decreased lung function and more at risk of respiratory disease and infections due to childhood exposure.
Climate changes, air pollution and allergic diseases in childhood and adolescence	Urrutia-Pereira,et al. (2022)	N/A	N/A	Air pollution	N/A	Respiratory outcomes- development of respiratory system	N/A	N/A	N/A	N/A	N/A	N/A	N/A	Both gaseous compounds (O3, VOC, NOx) and PM are well-established inflammatory stimuli to the mucosa of the respiratory tract. Depending on the aerodynamic diameter of PM, its action can occur at different points in the respiratory tract: PM10 is deposited in the nasal activity and central airways. Moreover, the action pollutants have on the cells of the immune system compromises the immune tolerance mechanisms and the antiviral and antibacterial defense mechanisms, making infections by respiratory agents more frequent.
Association between PM(1) exposure and lung function in children and adolescents: a systematic review and meta-analysis	Zong et al. (2022)	7	2006-2020	PM1	Short-term exposure (lasting less than 28 days)(n=3) and long-term exposure (28 to 91 days)(n=4)	Four lung indicators - FVC/FEV1/PEF/MMEF	Ages ranged 7-14 yr	N/A	China (n=5), Austria (n=1), Poland (n=1)	NOS	N/A	Overall, all of them scored higher than 7 points. Significant heterogeneity was detected in some of the analyzed PM1 and lung function indicator combinations	Very few studies included	Per 10 ug/m3 increase in PM1 exposure was associated with a decrease in indicators of lung function, in the short-term group. In the long-term group, per 10 ug/m3 increase in PM1 was associated with a decrease in indicators of lung function. Thus, both short-term and long-term exposure to PM1 results in decreases in four lung funciton indicators, which are commonly used to measure obstructive and restrictive lung disease. Exposure over a long period of time has a more pronounced effect on lung function compared to short-term exposure.
Adverse early-life environmental exposures and their repercussions on adult respiratory health	Mocelin,et al. (2022)	N/A	N/A	Air pollution	N/A	Respiratory health	Children, age not specified	N/A	N/A	N/A	N/A	N/A	N/A	Prenatal and early life exposure to air pollution can lead to impaired lung function, which remains largely unaltered until the sixth decade of life. These outcomes are related with late-onset respiratory diseases with reduced lung function. Children living near major highways have a lower FEV1 than those living further away. This reduction in FEV1 is associated with the development of COPD. Having severe childhood asthma was the strongest predictor of life of COPD at age 50. Persistently low or rapidly declining lung function and development of COPD are associated with risk factors present in childhood (maternal and paternal asthma, childhood asthma, respiratory infections). Moreover, reduced lung function at birth and less-than-expected increased lung function in early childhood are associated with an increased risk of respiratory disease. Children of mothers exposed to CO have lower lung function at 30 days of life, especially females.
Outdoor particulate matter exposure and upper respiratory tract infections in children and adolescents: a systematic review and meta-analysis	Ziou, et al. (2022)	34	1996-2019	Air pollution: PM10, PM2.5, fine PM, ultrafine PM	12, 19, 10, 1	Respiratory outcomes- upper respiratory tract infections (measured through ED visits, hospital admissions, in-patient visits)	Ages ranged from 0 to 18 years of age	Ranged from 40 to 3,472,347 participants	China (n=13), Europe (n=6), USA (n=5), Mexico (n=2), Australia (n=2)	Study quality assessment using NOS scale, and another standardised tool for case-crossover and time-series studies (score 0-5). Meta-analyses results reported at OR or RR	The RR values for PM2.5 and PM10 was 1.010 (95%CI= 1.007-1.014), and 1.016 (95%CI= 1.011-1.021). All were statistically significant. By visit type, ED visits (RR=1.017,95%CI: 1.010-1.24), hospital admissions (RR=1.037, 95%CI: 1.017-1.057), and outpatient visits (RR=1.009, 95%CI: 1.004-1.013) for URTIs increased with PM10 concentrations. For PM2.5, only outpatient visits was significantly increased (RR= 1.008, 95%CI: 1.004-1.011).	The quality of the 5 cohorts varied between 4-7 points out of 9. Cross-sectional studies obtained 8 out of 10. Case-crossover and time-series studies, 8 of them as of low quality, 14 of mediate quality, and 5 of good quality.	Due to small number of studies from different regions of the world, heterogeneity was not analyzed.	All studies reported a significant positive association between PM and the occurrence of URTIs in children and adolescents. The results obtained for PM2.5 and PM10 were more equivocal. Variation in designs, statistical methods, etc., However, the meta-analyses showed that positive small but significant associations were higher for PM10 than PM2.5. While PM2.5 is known to penetrate deeper into the respiratory tract than larger particles, and thus may have broader biological effects, larger particles tend to deposit in upper airways through impaction or sedimentation where they cause irritation, inflammation, increase epithelium permeability, and decreased mucociliary clearance, all of which reduce defenses against URTIs.

Effects of prenatal exposure to NO2 on children's neurodevelopment: a systematic review and meta-analysis	Shang, et al. (2020)	10	2012-2019	Air pollution: NO2	10	Neurodevelopment	Ages ranged from 6 months to 8 years of age,	Samples sizes ranged from 438 to 33,890 children.	Italy, Spain, China, Korea, Japan, The Netherlands, Germany, France, & Greece	Meta-analysis results reported using standardised exposure of 10 ug/m3 increase in NO2.	The effect of maternal NO2 exposure on children's language was -0.13 (95%CI: -0.34, 0.60). A 10 ug/m3 increase in maternal NO2 exposure was significantly associated with children's global psychomotor (-0.75, 95%CI: -1.34, -0.18).	Most studies rated a "low" or "probably low" in risk of bias. The heterogeneity for general cognition and maternal exposure to NO2 was median (I2= 48.1%). For language and maternal exposure to NO2 association was low (I2= 14.16%).	It was not conducted meta-analysis on attention, behavior, and IQ, as there were very few studies, and the measurement with behaviour and attention was inconsistent.	Prenatal exposure to NO2 has negative impact in neural development for children and this finding was biologically explicable. Moreover, by meta-analysis, only psychomotor function was found to be impaired. SOme studies have found that diesel exhaust particles, black carbon, or NO2 exposure during pregnancy decreased the motor function in the offspring mice, and this effect of diesel on the psychomotor was as well corroborated by a couple of human studies.
Children environmental exposure to particulate matter and polycyclic aromatic hydrocarbons and biomonitoring in school environments: a review on indoor and outdoor exposure levels, major sources, and health impacts	Oliveira,et al. (2019)	17	2007-2017	PM10, PM2.5, PM1, O3, and NO2	64%, 76%, 13%	School environment concentration of air pollutants	Children 3 to 18 years old	N/A	54% conducted in Europe, 26% in Asia, 16% in America, and 4% in Africa and Oceania	N/A	N/A	N/A	High heterogeneity in the geographical location, method design and exposure measurement	Evidence revealed that a strong contribution of indoor air quality (IAQ) of schools have on children total exposure to PM and PAHs. Children attending urban schools are at increased exposure of air pollutants and also present early markers of genotoxic damage and a lower capacity to repair DNA, leading to higher prevalence of chromosomal aberrations.
A meta-analysis of stressors from the total environment associated with children's general cognitive ability	Nilsen, et al. (2020)	185	N/A	While this meta-analysis included 408 maternal stressors, 245 inherent characteristics, 128 activities and behaviours, 125 social stressors, and 110 chemical stressors, the only relevant stressors for this review are toxic element stressors (e.g., mercury, lead), PAHs, NO2, phthalates, PCBs	25 (toxic elements), 16 (toxic gases), 5 (PAHs), 8 (Mn), 3 (Manganese), 11 (lead)	Children's cognitive abilities	Children	N/A	Primarily based in the US	The factor groups were analyzed using the random-effects meta-analysis with restricted maximum likelihood estimator. Output reported as odds ratio.	Toxic element stressors seemed to increased children's likelihood of suffering an impact on cognitive ability (OR= 1,04, p<0.011). Hg resulted in a 10% increase in impacting childhood cognition (OR=1.10, p<0.001), while lead had a 3% increase (OR= 1.03, p<0.001). Both Mn and As seemed to decrease the likelihood of impacting children's cognition (OR= 0.90, p<0.05; OR= 0.95, p<0.01, respectively). Toxic gases collectively increased 6% the likelihood of impacting children's cognitive abilities (OR= 1.06, p<0.001). PAHs had a 35% increase (OR= 1.35, p<0.001).	N/A	With such heterogeneous studies included, it could not report specific "positive/negative" relationships with certainty, and had to report the "likelihood of impact". Yet, the results reported in this meta-analysis are the synthesis of high-quality observational and randomized controlled trials that met the rigorous criteria set.	The toxic elements stressors showed to have a 4% increase in the likelihood of impacting childhood cognitive ability. The toxic gases showed to increase a 6% the likelihood of impacting children's cognitive abilities, with PAHs having the most effect or impact of all (35% increase).
Environmental influence on neurodevelopmental disorders: potential association of heavy metal exposure and autism	Ijomone, et al. (2020)	N/A	N/A	N/A	N/A	N/A	N/A	N/A	N/A	N/A	N/A	N/A	Small sample sizes and high heterogeneity in difference sampling approaches suggests to be cautious when interpreting the results	Excessive metal exposure has a detrimental effect on the nervous system. Neurons and glia in the developing brain are vulnerable to damage by metals such as lead and mercury which may results in permanent neurodevelopmental damage. Also, children have an immature and ineffective sequence of metabolism and detoxification, with poorer immune system especially during infancy. PB exposure is seen detrimental, particularly in early life. It has been named as one of the causes of ASD development. Mercury overexposure followed by the metal crossing the blood brain barrier and building up particularly in the cerebellum, visual cortex and the spinal cord. Several occurrences occur in the brain following mercury exposure which are similar to the brain of an ASD victim (e.g., neuroinflammation, dendritic overgrowth, mitochondrial dysfunction). Manganese is known to result in structural, functional and neurochemical alterations of the dopaminergic system.
The association between lead and attention-deficit/hyperactivity disorder: a systematic review	Donzelli et al. (2019)	17	N/A	Lead (pollution from lead)	Lead in blood (n=14), in urine samples (n=2), lead in molar teeth (n=1)	ADHD	N/A	Ranged from 117 to 2195, summing up to 8940 participants	Taiwan (n=3), South Korea (n=4), Germany (n=1), Spain (n=1), Belgium (n=1), China (n=1), Turkey (n=1), Mexico (n=1)	Not reported	N/A	N/A	Exposure during pregnancy or early childhood, no differentiation between age groups in the presentation of results. No quality assessment reported.	Lead levels were significantly associated with one of the types of ADHD (n=12). Other found no significant association (n=5).
Particulate matter exposure and attention-deficit/hyperactivity disorder in children: a systematic review of epidemiological studies	Donzelli, et al. (2020)	12	N/A	PM exposure (PM10 and PM2.5)	N/A	ADHD	Ages ranged from 3 to 14 years old	Sample sizes ranged from 2,618 to 66,283 participants	1 (Denmark, the NL, Germany, France, Italy Spain, Sweden), Germany (n=2), Japan (n=2), Korea, Spain (n=2), US, Belgium, Sweden, India	Quality assessment using the NOS scale (score 0-9)	N/A	All studies received a minimum of 7 in the quality assessment rating. Thus, can be considered at a minimum good quality studies.	One limitation is the heterogeneity of methodologies used in the studies, and variations in outcome assessment. Also, the various studies explored various windows of exposure, which contributes to their heterogeneity.	The results from 9 out of 12 studies indicated an increased risk of ADHD associated with PM exposure. It was found that ambient PM exposure is associated with attention disorders in most of the epidemiological investigations included in this review. Some studies also found that higher PM concentration levels tend to increase ADHD risk.

Assessing school children's exposure to air pollution during the daily commute- A systematic review	Ma, et al. (2020)	31	2004-2020	TRAP, PM2.5, PM10, CO, UFP, NO2, BC, PAHs (measured through personal monitoring, FMSs, air quality modeling)	N/A	Air pollution's effect on commute road	Schoolchildren	N/A	USA, Italy, Indonesia, Australia, India, UK, Mongolia, Canada, India, Spain, Korea, France, Iran, China, Ghana, Belgium	N/A	N/A	N/A	N/A	Two studies have reported that schoolchildren spend 4-5% of recording time for travel (commuting) but received 12% of BC and 10-11% of UFP exposure, respectively. Thus, commuting is a significant contributor to daily total air pollution exposure for schoolchildren. Moreover, in planning their routes, active commuting school children are encouraged to avoid major intersections as they are linked with exposure spikes; keep the distance from traffic queues; and avoid travelling along major roads with heavy traffic flow. In regards to car commute, the results were conflicting and mixed and thus, further investigation is needed.
The impact of traffic-related air pollution on child and adolescent academic performance: a systematic review	Stenson, et al. (2021)	10	2015-2021	TRAP: through either total length of all roads/major roads, estimated diesel PM health risk from on-road mobile sources, estimated load of traffic-associated PM2.5 around school, estimated ambient concentrations of PM2.5, PM10 and ozone	10	Academic performance - test scores, reading scores, and GPA	Schoolchildren	Outcome data ranged from 158 to 3,660 schools, 1,450 to 57,025 children	USA (n=9), The Uk	N/A	N/A	The overall risk of bias was rated to be serious. Main common source of bias was the use of poor quality exposure assessments of TRAP. Overall confidence in the level of evidence was low. All studies were observational, which according to the OHAT tool, already rates them as low.	The study designs were highly heterogeneous, making it difficult to identify consistent patterns of association for specific combinations of individual pollutants, age groups, exposure windows, and exposure locations.	Out of the 10 studies, 9 found some significant association between TRAP and academic performance. Children and adolescents exposed to higher levels of TRAP pollutants showed poorer academic performance than those exposed to lower levels of TRAP pollutants. However, the majority of analyses extracted in this review found null associations. Thus, the results are rather suggestive and cannot confirm any hypothesis of this association between TRAP and academic performance.
Air quality around schools: Part I - A comprehensive literature review across high-income countries	Osborne, et al. (2021)	100 records	N/A	Air pollution: NO2, PM2.5, PM10, O3, UFP	N/A	Air pollution levels around schools	School children	N/A	14 studies were UK-based, the others were in Spain, United States, Canada, New Zealand, Italy, France, South Korea,	N/A	N/A	N/A	N/A	Many of the studies observed that the journey to school can result in significant exposures to pollutants (e.g., black carbon), as well as UFP, and PM 2.5. Because inhalation rate is elevated during active travel, studies that calculated inhaled dose saw a contribution of up to 20% of personal exposure to Black carbon. Moreover, proximity to nearby roads was identified as a key factor influencing concentrations on school grounds, as well as the volume of nearby traffic. Also, higher greenness within and surrounding school boundaries were consistently associated with lower outdoor school concentrations. While sandy playgrounds and high density of surrounding buildings was associated with higher concentrations. Particulate levels outside schools were generally higher in colder months, NO2 levels outside schools were also observed in colder months. Diurnal patterns were also observed. Morning and evening concentrations peaks outside school corresponding to rugh-hour traffic were observed by many authors, with the morning peak typically the most extreme. For children walking to school, the choice of route is key and children walking on the quieter side of the road can avoid significant exposure.
Effects of air pollution on the nervous system and its possible role in neurodevelopmental and neurodegenerative disorders	Costa, et al. (2021)	N/A	N/A	Air pollution	N/A	Neurodevelopmental disorders (ASD, AD, and neurodevelopment in general)	N/A	N/A	N/A	N/A	N/A	N/A	The limitations of the studies included are heterogeneity in the characterization and quantification of the exposures, and inaccurate exposure estimations.	Exposure to air pollution may damage the developing brain and potentially contribute to neurodevelopmental disorders. Particularly, the evidence shows that air pollution plays a role in ASD development. In general, PM2.5 appears the most significant component of air pollution associated with adverse CNS outcomes, while NOx and O3 have shown more inconsistent results. UFPM is believed to be the most important PM for neurotoxicity, because of the biological characteristics which allows it to gain access to the CNS.
Air pollution and cognitive impairment across the lifecourse in humans: a systematic review with specific focus on income level of study area	Chandra, et al. (2022)	53	2007-2019	Multiple air pollutants, TRAP, PM, Isophone, POPs, PAHs	N/A	Cognitive health	Children and adolescents (up until 18 years of age)	N/A	US (n=21), Taiwan, Chile, Sweden (n=4), Netherlands (n=3), Uk (n=3), Spain (n=6), Canada, Germany (n=5), Italy (n=2), Poland, Mexico (n=5), China,	Study quality assessed using the Revised tool for Quality Assessment on Diagnostic Accuracy Studies.	N/A	Most studies scored high on the quality of primary diagnostic accuracy. The risk of bias was low to moderate. This indicated that the research on cognitive impact of air pollution is of good quality and can inform policy choices.	Exposure assessment varied among studies, which could be a limitation. Due to the heterogeneity in exposure and outcome variables, and tools used to assess these parameters, a meta-analysis could not be conducted.	Overall, the evidence indicates an association between exposure to pollutants and cognitive health effects across the life course. Most studies reported an adverse cognitive impacts of postnatal exposure to air pollutants, especially TRAP, POPs, and Isophorone. These associations were validated by cognitive testing, neuroimaging, and research on inflammatory markers. The plausible aertio-pathogenic pathways of air pollution's effect on cognitive decline included structural changes in brain, neurodegeneration, and neuroinflammation. There were no original studies conducted in LMICs, thus, the findings cannot be applicable to these countries.

Evidence of susceptibility to autism risks associated with early life ambient air pollution: a systematic review	Yu, et al. (2022)	19	2010-2021	PM2.5, PM10, NOx, O3, SO2, CO	11, 10, 12, 6, 1, 1	ASD	Prenatal and postnatal exposure	N/A	US (n=15), Sweden, Israel, Denmark, Canada.	NOS. The risk of bias was assessed according to the qualitative guidelines based on the GRADE principles.	N/A	All 17 observational studies passed the cut-off score of 7 for acceptable quality. The two ecological studies were excluded from this analysis. Most studies were rated as low in risk of bias, with few exceptions.	SOme of the limitations were small sample sizes to detect any significant effect, some selective reporting bias, and absence of standard assessment method. It led to not being impossible to conduct a meta-analysis	Most studies reported that the effect estimates of early life exposures of both PM10 and PM2.5 were stronger in boys than girls. There was a consistent pattern of larger air toxic effects sizes for boys than girls, as well as airborne lead was a risk factor for ASD boys but not girls in two studies. No significant or consistent pattern of effect modification on air pollution-ASD association was found by education level. Based on deprived or not neighbourhoods, no significant difference on the effect of air pollution-ASD was found. The same for genetics.
The effects of traffic air pollution in and around schools on executive function and academic performance in children: a rapid review	Gartland,et al. (2022)	9	N/A	TRAP: PM2.5, PM10, EC, BC, NO2, NOX, O3 , CO, PAHs	8, 4, N/A, N/A, 6, N/A, N/A, N/A,N/A	School attainment, through standardized test scores or grade point average	School children, ages ranged from 6 to 18 years of age	N/A	BREATHE project (Spain, Uk, Austria, Netherlands, Belgium), USA (3), and Chile (1)	NOS	N/A	The average score was 4.6, ranging from 2 to 7. Five studies rates as high quality. Papers that directly measured pollution levels, took repeated outcomes, and controlled for relevant variables.	The lack of compatibility across studies of the impact of TRAP on attention made it difficult to draw any firm conclusions. No studies reported an effect of NO2 or BC on attentional outcomes.	There is evidence to support the hypothesis that air pollution from traffic sources has a negative effect on both the executive function and academic achievement of primary-school-aged children. The relation between TRAP in and around schools and working memory becomes stronger when working memory is assessed over longer time periods. However, it is unclear from this review whether the effects of pollution act in particular developmental window or if the differential in the trajectory results of the cumulative effects of continued exposure to high levels of air pollution. The studies investigating the relation between air pollution and academic achievement support the suggestion that PM in particular has negative effects, while the evidence for the effects of NO2 and O3 is weaker. Taken together, the evidence relating to PM2.5 suggests that it affects both executive function and academic achievement and that the effects become stronger over time. Moreover, in summary, the limited evidence suggests that No2 has a specific effect on working memory and may not affect other facets of executive function.
In-utero exposure to air pollution and early-life neural development and cognition	Yi, et al. (2022)	12	N/A	Air pollution: PAHs, CO, NO2, PM10, PM2.5	2, 1, 4, 3, 4	Cognitive health- IQ, ASD, attention, verbal development	Prenatal and postnatal exposure	N/A	Spain (n=2), USA (n= 4), China, Mexico, Italy, Korea, Guatemala, Poland,	N/A	N/A	N/A	N/A	In-utero exposure to PAHs were associated with a 3% reduction in head circumference at birth, and reduced white matter on the left hemisphere in childhood. Most studies reported association between prenatal air pollutant exposure and lower IQ, as well as symptoms of depression, anxiety, and inattention at ages 6-8. Moreover, it has been linked to increased risk of autism in two studies, and to impaired psychomotor development between the first years of life. Furthermore, the studies showed that boys are more susceptible to the adverse effect of prenatal exposure to air pollution on cognitive and behavioural disorders. But females are more susceptible to the adverse effects of ozone on respiratory disorders. The potential mechanisms by which maternal air pollution exposure affects brain development is largely unknown. Yet, neuroinflammation has been recognised as the leading factor for neurological disorders.
Air pollution and neurodevelopmental skills in preschool and school-aged children: a systematic review	Castagna, et al., (2022)	30	N/A	TRAP: PM, NO2, EC, BC, PAHs	30, 13, 12, 4, 5, 6	Neurodevelopment - intellective functioning, learning and memory, attention and executive functions, verbal language abilities, numeric ability, motor	Ages ranged from 3 to 12 years of age	N/A	Spain (n=12), 3 Netherlands (n=2) Italy, Poland, Germany, Belgium, The UK, USA (n=9), Mexico, and Tehran.	Quality assessed using the Quality Assessment Tool for Quantitative Studies (score 1-3; 1 being the strongest quality).	N/A	12 studies scored 1, while 6 scored a 3 (weak quality). Thus, most studies could be considered of adequate quality scoring 1-2. Yet, a wide heterogeneity was reported regarding neuropsychological tools, geographical areas and sample size.	Limitations that limited the chance to carry out a meta-analysis were variability in the tools used for measuring the same neurological function or ability, estimation of air pollution focusing in particular geographical areas of certain countries, limiting the generalizability.	Prenatal exposure to PAH was negatively associated with mean scores of IQ points. Most studies revealed that several air pollutants were associated with inattentiveness in children of 6-11 years of age. Specifically, to TRAP, NO2, PM10, PM2.5, PHAs, and EC and poorer attention. Larger sample studies found that NO2 and PM2.5 are a risk factor for attention/execuitve functions at ages 6 to 11, specially in female children. PAHs, NO2, BC and PM2.5 were associated with learning and memory function decrease. Taken together, the results suggest that exposure to air pollutans is a potential risk factor for neurodevelopmental skill development in preschool and school children. Although verbal language, numerical skills, and sensorimotor abilities are affected by air pollution, the most adverse outcomes were on global intelligence functioning. executive functions and attention.
A systematic review of the mental health risks and resilience among pollution-exposed adolescents	Theron, et al. (2022)	17	1996-2020	Air pollution (ozone, NO2, NOx, PM2.5 and PM10), water pollution. It was measured in some as exposure for part of a day or a day, for up to 7 days, a month, a year, or longer.	11, 2. A DAY (N=2), up to 7 days (n=5), a month (2), a year (n=3), or longer (4)	Mental health- depressive symptoms, neurodevelopment symptoms, anxiety symptoms, and conduct disorders.	Adolescents (10-24)	Samples ranged from 40 to 75,643 participants.	Asia (n=5), Europe (n=4), USA (n=3)	GRADE	N/A	Studies showed methodological heterogeneity. Only 5 studies had samples that were nationally representative. Study quality varied, with seven studies rated moderate quality, eight low, and two very low.	Based on that all studies were observational limits the understanding of potential mechanisms linking pollution exposure with mental health outcomes.	Exposure to air pollution (traffic emissions, or ozone, NO2, PM2.5, PM10) was significantly associated with symptoms of depressed mood, generalised anxiety disorder, psychotic disorders, specifically delusions, hallucinations, unusual experiences, and poorer general mental health. Adolescents in the top quartile of air pollution exposure reported higher rates of psychotic experience. Moreover, adolescents in the top quartile of exposure to air pollution at age 12 were 3-4 times more likely to be diagnosed with depression at age 18 than adolescents in the bottom quartile. Except for one study, the studies that investigated associations between air-borne pollution and adolescent depression symptoms all reported positive associations. Lastly, studies did not explicate factors that protected the mental health of pollution-exposed adolescents.

Affective disorders and brain alterations in children and adolescents exposed to outdoor air pollution	Xie et al. (2023)	28	2008-2022	PM; Nox, O_3, EC, CO, PAHs, and lead	N/A	Anxiety/depre ssive symtpoms; bipolar symptoms; Brain changes (n=9),	3 to 18 yr	20 to 54,923	North America (n=13), Europe (n=10), Asia (n=5).	Quality assessment tool for observational cohort and cross-sectional studies of te National Institute of Health	N/A	Overall good quality with low risk of bias	High heterogeneity in exposure assessment	Air pollution increases the risk of depression and suicide-related events. In addition, neuroimagining studies revealed that exposure to air pollution i associated with alterations in the brain structure, function and metabolism, such as decreased white matter in the prefrontal lobe, parietal and temporal lobe (n=4).
Systematic review and meta-analysis of selected health effects of long-term exposure to traffic-related air pollution	Health Effects Institute (2022)	353	N/A	TRAP exposure in children	118	Respiratory outcomes,	Children, adults and elderly	N/A	Europe mostly (n=163), North America (n=130), Asia (n=41)	N/A	N/A	The overall confidence in the evidence for an association between exposure TRAP and asthma onset in children and ALRI in children was considered moderate to high	N/A	Summary estimates for NO_2 per 10 ug/m3 increase for asthma onset in children were 1.05 (95%CI: 0.99-1.12), and 1.09 (1.03-1.16) for ALRI in children.
The effect of noise exposure during the developmental period of the function of the auditory system	Bures, Z., Popelar, J., & Syka, J., (2017)	N/A	N/A	Environmental noise	N/A	Development of the auditory system	Children and animal testing	N/A	Not specified, but laboratory testing	N/A	N/A	N/A	N/A	The effects of early sound exposure (as seen in the experimental studies in animals) often persist to adulthood as permanent impairments of the hearing functions. However, many of their characteristics are different from those of acoustic overexposure performed in adulthood. Early exposure mostly blocks or retards normal development, conserving the auditory system in a premature state, which is characterized particularly by less precise frequency tuning, less specific targeting of inhibitory projections, and a larger total inhibitory strength compared to the adult system. However, studies studying the relationship of hearing impairment and behavior are relatively scarce. In regards to morphological alterations which can also appear, exposure to intense noise may cause significant damage to outer hair cells (OHC), inner hair cells (IHC) and their stereocilia, both in developing and adult animals.
WHO Environmental noise guidelines for the European Region: a systematic review on environmental noise and cognition	Clark, C., & Paunovic, K., (2018)	34	N/A	Traffic-related noise	13	Cognitive health- reading and oral comprehensio n, short-term and long-term memory, attention, and executive function deficit	Ages ranged from 8 to 12 years of age	N/A	N/A	Quality assessment using the GRADE methodology, which ranks the studies as high, moderate, low or very low.	N/A	Most studies rated (70%) rated as either low or very low quality, while only approximately 30% of studies were rated as moderate in terms of studies that evaluated road traffic noise exposure.	No meta-analyses was conducted due to the high heterogeneity in the designs and methods used in the studies for each cognitive ability domain, and exposure definition and assessment. Overall, the quality of the studies is very low, thus, the results cannot be interpreted as conclusive findings or inform policy-makers.	There was a moderate quality evidence across the available studies for an effect of aircraft noise on children's reading and oral comprehension. There was very low quality evidence for no substantial effect of road traffic noise on children's reading and oral comprehension.
Environmental noise exposure and neurodevelopmental and mental health problems in children: a systematic review	Zare Sakhvidi et al. (2018)	12	2001-2017	Road traffic noise (n=7), railway traffic (n=1), and aircraft noise (n=5)	Noise at school (n=5), residential noise (n=7)	Emotional symptoms (n=7), conduct problems (n=7), social adaptability (n=1), peer-relationship (n=6), hyperactivity/i nattention (n=6), anxiety (n=1), and mental health (n=2)	7 to 18 years	58,458-samples sizes of minimum 300	Germany (n=2), Norway (n=2), Denmark (n=1), Spain (n=1) Austria (n=1), EU (n=1), the UK (n=1), Macedonia (n=1), Bulgaria (n=1)	RoB with the modified checklist by Dzhambov and Dimitrova; Quality by GRADE	N/A	Seven studies had high quality score, and five had moderate of RoB. According to the GRADE system, the overall quaity was low to very low.	Very limited studies with high heterogeneity, the interpretation of the association is only suggestive	An increased risk of conduct problems was observed with traffic noise (n=4), an increased risk of hyperactivity/inattention problems (n= 2 out of 3), and with inattention at ages 3 and 8 (n=1). On mental health, none found a significant association. The results of the studies on nightime noise exposure and at home residence showed higher significance compared to daytime noise and at school settings. The authors suggests that it could be because: 1) children spend less time on school compared to home; 2) they might pay less attention to noise during daytime (because they can be playing) than at night.
Behavioral and emotional disorders and transportation noise among children and adolescents: a systematic review and meta-analysis	Schubert, et al., (2019)	14	2001-2018	Noise (railway and road noise)	14	Mental health- behavioural problems, peer relationship problem scores, total difficulties, depression, anxiety, emotional symptoms	Children and adolescents of ages 9 to 17 y/o	N/A	Asia, the rest of studies in Europe and UK	Quality assessment using a hybrid tool that combined characteristics of the SIGN and CASP assessment tools. Meta-analysis conducted using standardized estimates of exposure.	Significant association between road traffic noise, hyperactivity/inattention (OR= 1.11; 95%CI: 1.04-1.19), and total difficulties (OR= 1.09; 95%CI: 1.02-116).	Unexplained heterogeneity for the outcomes hyperactivity/inattention, emotional symptoms and total difficulty scores.	N/A	7 out of 9 studies found a significant positive association with transport-related noise exposure. Findings regarding risk for emotional or behavioural problems and exposure association were inconclusive. Overall the evidence was reported as moderate, and it indicated that exposure to traffic noise was associated with conduct disorders. 2 studies reported noise-related sleep disturbances which may increase risk for hyperactivity problems.

Noise pollution and human cognition: an updated systematic review and meta-analysis of recent evidence	Thompson, et al. (2022)	16	2015-2021	Mixed noise pollution (railway, aircraft, and traffic noise)	12, 1, 2,	Academic performance; reading, verbal and language ability; attention, executive function, memory, fluid intelligence	Children, newborns and adults	N/A	US (n=9), Europe (n=29), Asia (n=4), & Africa (n=2)	Risk of bias was assessed using the OHAT Risk of Bias Rating Tool. Overall quality assessed using the GRADE methodology (score 1-3).	Significant higher reading test scores in quieter classrooms (OR= 1.80; 95%CI: 0.40-1.20).	Significant heterogeneity in the meta-analysis (p<.001). Quality of evidence generally very low. Downgrading occurred mostly to risk of bias concerns and inconsistency with findings.	The meta-analysis cut across multiple outcome metrics, which only demonstrates the trends of effects rather than precisely estimating exposure-outcome relationship.	Children attending schools with low noise show better math and reading test scores compared to children at schools with higher traffic noise. The evidence did not support an association between traffic noise exposure and language and reading abilities.
Environmental noise exposure and emotional, aggressive, and attention-deficit/hyperactivity disorder-related symptoms in children from two European birth cohorts	Essers,et al. (2022)	A meta-analysis of two birth cohorts	From 2015 to 2022	Prenatal traffic noise by home address exposure	N/A	Emotional, aggressive, and ADHD-related symptoms	Birth cohorts	10,257	Sweden, and Spain	No tool to assess quality of studies	No association between prenatal road traffic noise exposure and emotional, aggressive, or ADHD-related symptoms in neither cohorts, separately or combined in the meta-analysis.	N/A	Detailed in the next column	They found no evidence of an association of residential road traffic noise exposure during pregnancy or childhood with emotional, aggressive and ADHD-related symptoms in children from two European birth cohorts. Associations were also absent from total noise exposure in which railway, aircraft, and industry noise exposure were additionally assessed. They also found an unexpected protective association between road traffic and total noise exposure and emotional symptoms at 9 years in children from the Generation R study. Of their population at generation R study at 18 months, around 26% had missing noise exposure levels at 9 years, because they moved outside Rotterdam and noise exposure could not be estimated. These children had parents with higher socio-economic status and reported less emotional symptoms at younger ages and more symptoms at 9 years old compared to those children who continued living in Rotterdam. Thus, their unexpected results were most probably due to selection bias.
Children's blood pressure and its association with road traffic noise exposure - a systematic reivew and meta-analyssi	Dzhambov et al. (2017)	13	N/A	Road traffic noise	Noise exposure at school/kindergarten and at home (n=7), noise at home (n=4), and at school (n=2)	Blood pressure	Ranged from <7 to >10 years	Ranged from 1542 to 115	Eastern Europe (n=4), Western Europe (n=6), Asia (n=2), USA (n=1)	For RoB they used a predefined quality checklist	Effect estimates for the change in BP per 5dB increase in RTN were pooled. Meta-analysis showed non-significant increase of 0.48 mmHg in systolic blood pressureand 0.22 mmHg in DBP per 5dB increase in noise levels at school/kindergarten, and non-significant increase of 0.20 mmHg in SBD and 0.03 mmHg in DBP per 5 dB increase in noise levels at home.	Heterogeneity in the four models was high. And the quality of the four meta-analysis would be graded as low.	The studies contained high risk of bias and the meta-analysis were considered low. Also, the heterogeneity in the geographical locations and in the exposure assessment make the authors rate the meta-analysis as low quality. Also, sensitivity analysis identified several effect modifiers, such as outcomes scenarios, and the country in which it was assessed.	Overall, the associations between RTN exposure and children's BP is non-significant yet positive associations were found at the school/kindergarten. In regards to home exposure, the increase in blood pressure was very low and also, non-significant.
Pediatric thermoregulation: considerations in the face of global climate change	Smith, C. J., (2019)	N/A	N/A	Heat	N/A	Thermoregulation and health	Children	N/A	N/A	N/A	N/A	N/A	N/A	Among the various morphological, physiological and psychological differences, the most significant are the following according to the review. Children have smaller total body surface area, higher metabolic heat production during exercise. Obese children have higher adiposity which may contribute to hindered heat loss, and they typically have lower maximal aerobic capacity, thus, they achieve higher heat production than non-obese children. Children have also less efficient locomotion than adults, leading to more heat produced and their resting metabolic is also higher than adults.
Heat-health vulnerability in temperate climates: lessons and response options from Ireland	Paterson, S. K., & Godsmark, C. N., (2020)	15	N/A	Heat	15	heat vulnerability on overall health	All populations, yet, with an individual section on children	N/A	Ireland	No tools used to assess quality of studies	N/A	N/A	N/A	Physiologically, there is a debate whether children are vulnerable to heat stress through underdeveloped and reduced sweating responses, reduced locomotion economy resulting in greater heat production per unit of body weight, and smaller total blood volume. Behaviourally, it is generally accepted that children are vulnerable to heat stress due to their decreased awareness of fluid intake requirements, spending time outdoors in play as well as often playing on, or close to the floor where thermal radiation levels can be high depending on solar intensity and surface material.
Global warming, heat-related illnesses, and the dermatologist	Williams, M. L., (2021)	N/A	N/A	heat	N/A	thermoregulation differences on child versus adults	children and adults	N/A	N/A	N/A	N/A	N/A	N/A	Large differences between children and adults in thermoregulation include a greater deposition of subcutaneous fat in most infants which leads to accumulating more heat, higher metabolic heat rate during exercise, lower cardiac output at a given metabolic rate, lower volume of sweat produced per gland, and lower sodium content in sweat. Also, children and adolescents may not be aware of the importance of rehydration, consuming less water.
Associations between weather conditions and physical activity and sedentary time in children and adolescents: a systematic review and meta-analysis	Zheng et al. (2021)	26, 8 for the meta-analysis	2008-2020	Weather conditions and temperature	26	Physical activity	3 to 19 yr	43,211 participants	Europe (n=8), North-America (n=12), Australia (n=3), Asia (n=3), international (n=2)	NOS	Results reported as correlation coefficient ® Temperature was positively but not significant associated with PA (r=022; 95%CI: 0.09-0.35)	Overall, moderate to high quality	N/A	The majority of studies reported positive associations between temperature/maximum temperature and PA. The average temperature ranged from -12 in Alberta, Canada to 22 in Hong Kong. Moreover, several studies reported an inverse U-shaped relationship between PA and temperature where PA increased up to 20-25°C and then declined. One study reported associations between temperature and MVPA in Hong Kong, a city with exteme hot weather. Namely, children and adolescents reached optimal PA when the temperature was around 20.

Effects of regular classes in outdoor education settings: a systematic review on student's learning, social, and health dimensions	Becker, et al. (2017)	13	N/A	Green school environment as educational setting	13	Learning dimensions and social dimensions	Schoolchildren and adolescents	Average number of participants: 62.17. Ranged from 5 to 230 children and adolescents.	Denmark (n=4), USA (n=3), Germany (n=2), NZ, Sweden, UK, and Norway.	Quality assessed using the Child Care and Early Educational Research Connections Qualitative Research Assessment Tool. The quality of qualitative studies assessed using the JBI checklist for Qualitative Research.	N/A	High heterogeneity in regards to the design, methods and instruments used, learning environments and measured outcomes, as well as sample sizes. Methodological quality of quantitative studies was classified as low to moderate, and one study rated as high. For qualitative studies, they were classified as moderate, and two studies as high.	Several studies included were weak in terms of internal structure and given information. Very few limited studies with high heterogeneity that makes it hard to draw conclusions on the findings.	Participating in outdoor classes seem to benefit the students of improved academic performance, improved skills in transferring knowledge, improved learning motivation, improved social competences, self-esteem, and sense of belonging. As well, the studies reported positive attitudes towards the environment. Moreover, studies evaluating PA levels when participating in outdoor classes was underrepresented, yet, it showed to increase their PA levels.
Does access to green space impact the mental well-being of children: a systematic review	McCormick, R., (2017)	12	N/A	Greenspace exposure (through NDVI, land use/cover, proportion of green space, distance to green space)	12	Cognitive development	Children: 0-18 years	N/A	USA, Spain	N/A	N/A	N/A	N/A	Access to green space can improve mental well-being of children. Moreover, students attending outdoor classes and playing in wooded playgrounds, and natural habitats seem to find relief from stress, build confidence, and improve their focus. 3 studies found that high quality and high quantity of green spaces is associated with less total difficulties, improved child well-being, and less peer relationship problems. 4 studies found that green outdoor settings can reduce hyperactivity and inattention problems when being exposed, and it promotes attention restoration, improve behaviour and it is associated with higher test scores in various subjects.
Determining factors in the use of urban parks that influence the practice of physical activity in children: a systematic review	Padial-Ruz, et al. (2021)	31	N/A	Green space- parks	31	Physical activity levels	Ages ranged from 0 to 17 y/o	131,607 children	USA, Australia, Brazil, Spain, UK, The Netherlands, Denmark, New Zealand, Sweden	Methodological quality was assessed using Cohen's Kappa statistical index (Kc)	N/A	N/A	The data obtained is not highly consistent due to the scarcity and variety of studies.	Family and caregivers support the notion that parks can benefit children on social, physical, motor and cognitive skills. However, they highlight that user opinions are not taken into consideration when designing parks for increasing their use. According to the users, for children aged 0-6 y/o, parks should offer opportunities for outdoor activities, allowing for games that motivate children in a safely manner, in green outdoors and be close to home. Yet, family members and caregivers of children aged 7-12 y/o prefer larger parks with amenities (e.g., bathrooms, fountains)and sport facilities to allow for groups to exercise of different ages. This preference for large parks was seen also in children aged 8-12 y/o. Thus, parks must facilitate the practice of PA, allow space for community events, contain swings, climbing equipment, and other to allow for games in nature to be incorporated. Importantly, the diversity and accessibility are crucial factor to increase the use of parks, allowing different groups and genders to participate in activities in the same environment needs diverse spaces for different purposes.
Mental, physical, and social health benefits of immersive nature-experience for children and adolescents: a systematic review and quality assessment of the evidence	Mygind et al. (2019)	84	2004-2018	Immersive nature-experienced: Based camp adventures, green educational activities, outside classrooms, free play outdoors	N/A	Mental health, self-esteem, self-efficacy, self-concept, resilience, problem-solving, academic performance, cognitive performance, mood, physical health, BMI, social health, relation indicators	Range from 7 to 18	N/A	America (50%), Europe (30%), Oceania (14%), Asia (8%)	GRADE; RoB from inspiration of the Cochrane Collaboration's tool for randomised trials	N/A	Most studies included considered high risk of bias and low quality.	No meta-analysis due to heterogeneity of outcomes and interventions. The review analyses many outcomes and many different exposures, from 10-day science camps to 3-month trip to the mountain to outdoor classes. Include general populations, low SES populations and with mental problems.	Following immersive nature-experience, mental health outcomes were improved (n=23 out 35); improved participants' self-esteem compared to control groups (n=8 out of 11); led to increases in self-efficacy (n=5 out of 7). Cognitive performance was enhanced after walking and after various types of PA in natural environments (n=3). Improved concentration and memory (n=1) and improved mood (n=1) after outdoor classes. **Overall, across all outcomes, 60% (n=56) were improved upon immersive nature-experiences compared to control conditions**.
The associations between green space and adolescent mental well-being: a systematic review	Zhang, et al. (2020)	14	2013-2019	Green space exposure, measured either though Normalized Difference Vegetation Index (NDVI) approach, or real time global position system (GPS) technology, or through subjective experience of green space measure.	14	Mental wellbeing, variables were mood, stress, depression, emotional wellbeing, mental health, and behaviour and psychological distress.	Children and adolescents: 10-18 years old	Study sample ranged from 60 to 17,249 participants	2 NL, 2 UK, 5 USA, Canada, Germany, Austria, Australia, and NZ.	Lachowycz and Jones methodological quality assessment tool.	N/A	All studies scored equal to or above 7/11. Most were deficient in consideration of type of green space, no measure of green space or analysis at ecological level rather than individual.	No meta-analysis was conducted due to the high heterogeneity of green space and mental health outcome measures. Another issue that may limit the findings or their interpretation is that there is no appropriate and common measure that represents green space, and what can be considered green space.	Exposure to green spaces seem to benefit adolescents in terms of reduced stress, better emotional well-being, less symptoms of depression, improved mental health, and lower stress. Moreover, school-related green exposure evaluated in two studies found that widow views of green landscape was associated with lower stress and increased positive effects. Yet, issues of quality and access for all is of concern.
Landscape of becoming social: a systematic review of evidence for associations and pathways between interactions with nature and socioemotional development in children	Mygind, L., Kurtzhals, M., Nowell, C., (2021)	26	2001-2018	Residential greenery, through NDVI, or distance to nearest green space	26	Child emotional function and development	children over 6 years of age	N/A	Belgium, Denmark, Germany, Italy, Lithuania, The Netherlands, Spain, Sweden, UK, Canada, USA, Australia, NZ, South Korea, Israel, Iran	N/A	N/A	N/A	Due to the high heterogeneity of study designs, outcome and exposure measurements, a meta--analysis was not conducted. They identified few studies without risk of bias.	Exposure to natural environments was found to be associated with children's ability to form relationships, socially adaptive behaviours, overall socioemotional adaption, and lower risk for ADHD and ASD. Moreover, children playing in natural habitats seemed to engage more deeply in play than in other environments. No consistent associations between green exposure and obesity or overweight prevalence were found. Moreover, some evidence suggested that interaction with green spaces seem to improve motor skills, language and communication skills.

Psychological impacts of "screen time" and "green time" for children and adolescents: a systematic review	Oswald, et al. (2020)	186	2000-2019 (with 2018 having the most published)	Screen time (time engaged on visual screen-based technologies) and green time (incidental exposure to green space, accessibility to green spaces, purposive use of green space, or educational contexts in green spaces).	114, 58, 14(both exposures)	Psychological outcomes- mental health, cognitive functioning, and academic achievement	Young children (<5 y/o), school children (5-11 y/o) and adolescents (12-18y/o)	Ranged from 11 to 388,275 participants (median of 969)	The US (n=58), the UK (n=37), Australia (n=25) and New Zealand, Canada (N=17), and Europe (N=61)	N/A	N/A	N/A	The longitudinal studies were difficult to compare. A lack of consistency in the conceptualization and measurement of screen time limits compatibility between studies, which could ultimately make broader conclusions.	Many cross-sectional studies reported associations between ST or GT exposure with some, but not all, of the psychological outcomes. What is clear is that higher ST tended to be associated with unfavourable psychological outcomes while increased GT tended to be associated with favourable psychological outcomes. T Some longitudinal studies do confirm favourable relationships between GT and psychological outcomes, building the case for causal linkages. Moreover, it is not clear from this review what type of green time exposure is the most beneficial.
Life course nature exposure and mental health outcomes: a systematic review and future directions	Li, et al. (2021)	29	2007-2019	Life nature exposure, through either NDVI or others	29	Mental health outcome- mental disorders, psychiatric symptoms, behavioural problems, cognitive function, and subjective well-being	Prenatal exposure and children, and adolescents	Ranged from 45 to 943,027 participants	Europe (n=18), Australia (n=4) and New Zealand, North America (n=4), and Asia (n=3).	Risk of bias assessed using the Study Quality Assessment Tool developed by the NHLBI.	N/A	N/A	Due to the heterogeneity of exposure assessments, outcome measurements, study designs and populations, it remains inconclusive whether there is a critical window for nature exposure. As well, due to high heterogeneity a meta-analysis was not possible.	27 studies found a positive significant relationship between early nature exposure and a mental health outcome. No study found a detrimental main effect of early life nature exposure, and one article found an interaction effect where those who visited nature less often in childhood benefited more from taking nature visits in adulthood. Four studies found a protective role in nature exposure against schizophrenia; and for ADHD, higher residential grenness was associated with decreased risk in children and adolescents.
Greenspace and health outcomes in children and adolescents: a systematic review	Ye et al. (2022)	140, and 10 for meta-analysis	2007-onwards	Greenspace: NDVI, greenspace coverage, proximity to greenspace, perceived greenspace, greenspace visits, street view vegetation index	85, 58, 32, 12, 5, 4	Mental health (n=53), nutritional status (n=36), allergic and respiratory outcomes (n=28), circulatory health (n=10), other (n=5).	N/A (not reported)	N/A	North America and Europe (n=104), China (N015), Australia (n=12), South Korea (n=12), Nepal (n=1), Iran (n=2), Argentina (n=1)	RoB with Navigation Guide framework	Reported associations (RR, OR, or correlation coefficient). No significant association between asthma and NDVI (OR= 0.94, 95%CI: 0.84 to 1.06). No significant association with NDVI and AR (OR= 0.95, 95%CI: 0.73 to 1.25)	Significant heterogeneity for NDVI and Asthma (I2= 86%, p-value <0.01).	They report that the measurement of greenspace exposure is highly heterogenous between studies, and the lack of a clear definition of greenspace. Not able to perform a meta-analysis for each outcome-exposure pair due to the heterogeneity of the studies. **Unable to stratify results according to age groups due to lack of study numbers**.	Most epidemiological studies observed a statistically significant association between greenspace exposure and **mental health.** Null associations were also observed by some studies, where neighbourhood greenspace was neither protective nor promoted cognitive development or mental health. Results on **asthma** were inconsistent. Some reported a higher risk of asthma (n=3), and **childhood wheezing** and **AR** (n=1); other reported protective effects (n=8). In association with lung function, some observed better **lung function** and reduced impairment with higher greenspace (n=2). Studies reported significantly beneficial effect on **blood pressure** and **lower odds of hypertension** (n=3) with higher greenness surrounding schools; helped mantain **normal BP** around schools and home green space (n=1). And **increased PA** (n=1)and and **reducing CVD risks** (n=1).
Associations between nature exposure and health: a review of the evidence	Jimenez, et al. (2021)	N/A	N/A	Nature exposure	N/A (NR)	Cognitive and cardiovascular health and sleep	Children (<10 y/o), and youth (10-24 y/o)	N/A (NR)	Primarily Western Countries	N/A	N/A	N/A	The lack of studies on children and youth on green space exposure and affective state limit any possible generalizability. Methods to measure exposure are inconsistent among studies and lack standardization.	Blood pressure measured in forest environments was significantly lower than in non-forest environments. Natural features near schools is linked to pre-scholars improvement in socio-emotional competencies, decreased prevalence in ASD, and higher levels of PA. Some studies have shown that exposure to green space is associated with lower prevalence of obesity in children. Yet, most studies included regarding obesity and children showed mixed results. Recent evidence has found that exposure to residential green space is associated with decreased CVD incidence. Although limited evidence, it is seen inverse associations between time spent in green space and fasting blood glucose levels and insulin resistance. The relationship between exposure to nature and asthma and allergies is inconsistent,
Greenspace exposure and children behaviour: a systematic review	Zare Sakhvidi, et al. (2022)	29	2015-2022	Exposure to greenspace (based on either accessibility, availability, proximity, and quality) mostly at residential locations	29	Behavioural outcomes - ADHD, conduct problems, prosocial behaviour, emotional symptoms, peer-relationship disorders, and internalizing/externalizing disorders.	Age: from birth to 18 y/o	1,234,721 children, ranging from 169 to 814,689 children	Europe (n=14), the US (n=8), Asia (n=3), and Oceania (n=4)	Risk of bias assessed through the NOS scale.	N/A	Overall quality of the studies was fair (average score of 5.4 out of 9), with 2 studies with very good quality.	Due to high heterogeneity, they were unable to perform a meta-analysis.	The reported findings were suggestive of the potential association between greenspace exposure and reduced risk of behavioural problems. All six studies on green space exposure and internalizing/externalizing problems reported associations suggestive on the beneficial role of green exposure. Two-third of the studies on peer relationship problems suggested a protective role of green exposure on this outcome. More than half of the studies on emotional problems were suggestive of the beneficial role of green exposure. Most of the studies were suggestive on a protective role of green exposure on ADHD symptoms. However, no association was statistically significant. Two studies found that the effects of green space exposure were more significant at younger ages. The effect of green space exposure could be moderated by factors such as SES, sex, and ethnicity.
Exposure to greenspace and bluespace and cognitive functioning in children - a systematic review	Buckzylowska et al. (2023)	39	2000-2022	Green and blue spaces	Greenspace (n=39): NDVI, distance to greenspace; Greenspace and blue space (n=3)	Attention contorl (n=12), processing speed (n=10), working memory capacity (n=11), Visual processing (n=2), Intelligence (n=9), cognitive development (n=2), decision-making (n=3)	Children (0 - 10 Yr) (n=15), adolescents (n=5), both (n=17)	17 to 27372	Europe (n=27), North America (n=9), Asia (n=2)	AMSTAR-2; RoB with OHAT	N/A	none were rated as low risk, Four studies had high risk and eight had probably high risk.	N/A	A 20-30 min nature walk was associated with improved attention (n=1), faster reaction time)n=1), improved executive attention (n=2). Faster reaction time (n=3), inattentiveness (n=2), attention (n=7) and presence of greenness at school. Working memory capacity (n=5) and negative associaiton (n=4), improved working memory (n=3), visual processing: 2 found null associations, 1 found positive. 5 studies reported improved intelligence and natural environment, 4 no association. with childhood development (n=1). The only 3 studies with bluespace found no association with any of the cognitive domains.

The relationship between greenspace exposure and psychopathology symptoms: a systematic review	Tran, I., Sabol, O., & Monte, J., (2022)	35	N/A	Exposure to greenspace (based on either accessibility, availability, proximity, and quality) mostly at residential locations or work/schools	35	Psychopathology- mental health disorders and symptoms of mental health disorders	Age: from newborn to 85 y/o	Approximately 2,011,934 children and adolescents (aged newborn to 18 y/o)	Europe (n=20) and North America (n=15)	N/A (NR)	N/A	N/A	Methods varied widely in terms of exposure metrics, assessment and symptoms, and sample sizes. Due to this high heterogeneity, it could not be compared greenspace exposure effects on adults and on children.	Objective greenspace exposure was negatively associated with incidence of an ADHD diagnosis and symptoms in formally diagnosed children. However, it is unclear whether greenspace exposure is associated with less severe symptoms of hyperactivity and inattention. Two studies found that more green coverage was associated with less internalizing/externalizing problems in children. Some studies suggest that the relationship between green space exposure and psychopathology symptoms may be mediated by sociodemographic factors. Two studies found that children that lived in highly populated areas with more green coverage experienced less symptoms of depression than children living a less green coverage.
Does exposure to greeness improve children's neuropsychological development and mental health? A navigation guide systematic review of observational evidence for associations.	Luque-García, et al. (2022)	34	2002-2021	Exposure to green space (through NDVI, % of grenness or TCC)	34	Neuropsychological development and mental health	Prenatal exposure, children and adolescents until 18 y/o	Sample ranged from 169 to 59,754	Europe (n=17), the US (n=9), Australia (n=4), South Korea (n=2), and Canda and China.	Assessed quality and strength of the evidence through the Navigation Guide methodology, which integrates the GRADE methodology to assess risk of bias.	N/A	Most of the studies on neuropsychological domains were rated as very low quality, while the remaining on academic performance were rated as low quality. The studies on mental health were rated as moderate, low and very low.	Data not suitable for meta-analysis due to high heterogeneity in assessing exposure to greenness and in method design	Children whose home is surrounded by natural features showed larger tissues volumes in various brain regions compared to children living in homes with less vegetation. Also, the studies observed higher scores in self-discipline, improve in cognitive development, fewer ADHD symptoms, and fewer behavioural problems. Children attending schools with more greenness presented improved well-being, better academic performance compared to those attending schools with less tree cover. Moreover, children living in urban areas with more greens seem to benefit more than those living in rural areas with more grenness as urban children are more likely to take active transport on their route to school, the authors suggest. Lastly, in most studies, the associations between exposure to grenness and the domains studies remained significant after adjusting for SES characteristics.
Toxicant exposure and the developing brain: a systematic review and functional MRI literature	Fowler et al. (2023)	46	2000-2022	Air pollution	19	Brain structure - MRI	<1 to 14 yr	30 to 9697	Western Europe (n=19), USA (n=16), Mexico (n=5), South Korea (n=2), Taiwan (n=2), China (n=1)	NIHQAT for observational and cross-secitonal studies	N/A	Most studies were of moderate quality 65%), with few of high quality (22%)	Could not include results of metal exposure given that the source of metal is not clearly distinct in the results section	First, regardless of age of exposure or age oat MRI, summative measures of volume, thickness, and surface area did not seem to be associated with air pollution exposure. Late middle childhood exposure was not associated with white matter volumes in young adolescence or late middle childhood. Based on other findings from this review, the authors note, that air pollution likely impacts different brain regions, creating a null additive result. Also, given that most studies had a exposure measre and brian MRI and a very large time lapse between, it is possible an effect exists but these two measures need to be conducted in closer time. Yet, air pollution exposure in late middle childhood was associated wit reduce basal ganglia volume.
Air pollution and human cogniton: a systematic review and meta-analysis	Thompson et al. (2023)	86	N/A	PM2.5, Nox, PM10	36	General cognition (n=8); Intelligence/IQ /reasoning (n=9); Attention, working memory, and executive function (n=18)	0-18 yr	N/A	Europe (n=34), North America (n=32), East Asia (n=10), South America (n=2), Australia (n=1), Southeast Asia (n=1), International (n=1)	RoB with the NTP-Office of Health Assessment and Translation (OHAT) approach	N/A	Overall, the evidence was rated as low to moderate. Most studies raised some concerns of bias.	Studies included have low quality of evidence, for the majority.	General cognition: for PM2.5, the relation was generally unsuportive but with low certainty, and for Nox and PM10 as well. Intelligence/IQ/reasoning: mixed results without a clear and conclusive direction of evidence. Attention, working memory, and executive function: EC/BC was detrimentally associated with executive function skills, specially for working memory (n=8). Ten studies provided moderate certainty evidence supporting that PM2.5 was detrimental to executive function skills.
Residential greenness and allergic respiratory diseases in children and adolescents -- a systematic review and meta-analyses	Lambert. et al. (2017)	15	N/A	Green space exposure. Exposure measurements included street tree density, LiDAR imagery, and NDVI.	15	Allergic respiratory diseases: asthma, allergic rhinitis,	Age: newborn to 18y/o	Approximately over 781,222 participants in total	Europe (n=5) (Spain, Germany, Lithuania, Sweden, Germany), US (n=4) and Canada, Australia	A validated quality assessment framework was adapted to assess and rate the design, execution, generalisability, and risk of bias. The overall risk of bias assessment was guided by the GRADE methodology. Meta-analysis conducted with 95% confidence interval.	The meta-analysis of the three studies using the same measure of residential greeness exposure (NDVI) showed no significant overall association between asthma and residential greeness (OR= 1.01, 95%CI: 0.93-1.09). Moreover, the meta-analysis studying residential greeness exposure and allergic rhinitis on the studies with same exposure measurement did not found either a significant association.	Overall, methodological quality was high, with most studies scoring >75%, in one study, sample size was too small to detect a meaningful difference. The results of the two meta-analysis was significantly heterogeneous (I2= 68.1%, p value= 0.02; I2= 72.9%, p value= <.01)).	A key issue in these studies was the assessment and definition of the outcome. For asthma/AR the assessment varied from parental or self-report, to administrative records to clinical assessment. Moreover, the limited number of studies did not allow the authors to break the outcome variables further.	They found eight articles showing a protective effect of residential greeness, two studies showing a detrimental effect and food studies reporting no association. The meta-analysis showed no significant overall association between residential grenness and asthma, but it was highly heterogeneous. In regards to allergic rhinitis, variable effects were found again, and the meta-analysis showed no significant overall association between residential grenness and allergic rhinitis.
Influence of residential land cover on childhood allergic and respiratory symptoms and diseases: evidence from 9 European cohorts	Parmes, et al., (2020)	Meta-analysis of nine birth cohorts	N/A	Residential land cover: green space, grey space, blue space, agricultural space, forest, mixed forest	Meta-analysis of 9 cohorts	allergic and respiratory diseases- lifetime wheeze, current wheeze, lifetime asthma, current asthma, lifetime allergic rhinitis, and eczema	Age: 3 to 14 y/o	8064 children	European cohorts (n=9)	Statistical analysis reported using odds ratio with 95% confidence interval.	Proportion of green space covered land was significantly associated with increased odds of lifetime and current wheezing (+5.9% and +13% respectively, per 10% increase in green cover), as well as lifetime and current asthma and allergic rhinitis (+9.2%, +12.1%, and 8.1% respectively). Associations between green space and health outcomes were similar in males and females and across different ages groups. Children with forest near their home had increased odds of having current eczema (OR= 1.26, 95%CI: 1.05-2.97).	Significant heterogeneity between studies of association between green space and lifetime wheezing.	Difference in conceptualization of green space, age differences and sample sizes and designs show high heterogeneity which could limit the interpretation of the findings.	No statistically significant associations found between urban grey and blue spaces and any health outcome in the meta-analyses. The results of this meta-analysis suggests that allergic and respiratory symptoms can increase with increased nearby green space and are consitent with many, but not all, studies focused on similar associations. It could be because green space are sources of pollen, moulds, and aerosols, which creater allergies and respiratory problems. No associations found for urban grey or blue spaces for any of the respiratory outcomes. When evaluating agricultural space, these results show a moderately protective effect for all the respiratory outcomes, although not statistically significant.
Influence of residential land cover on childhood allergic and respiratory symptoms and diseases: evidence from 9 European cohorts	Hartley, et al. (2020)	7	2017-2019	Green exposure (e.g., NDVI)	N/A	Respiratory outcomes- Asthma	Children	Sample ranged from 150 to 49,956 children	USA, New Zealand, Australia, Spain	Quality assessment through the Johns Hopkins Research Evidence Appraisal tool	N/A	N/A	Difficulty in isolating the effect of grenness from other health-related factors such as tobacco smoke exposure, socioeconomic status.	Overall, 6 out of 7 reported no statistically significant direct relationships between greenness and child asthma while 1 found that greenness was associated with a 6% lower risk of asthma. 3 papers reported that grenness was protective for child asthma by mediating other negative health-related factors (i.e., difficult family relationships, and tobacco smoke exposure). Another study reported that children living in heavy traffic areas with low greens had a higher risk of asthma (OR: 1.87, 95%CI: 1.37-2.55), and those living in high traffic and high grenness had lower risk of asthma (OR: 0.32, 95%CI: 0.12-0.84).

Exposure to urban greenspace and pathways to respiratory health: an exploratory systematic review	Mueller, et al. (2022)	108	2007-2022	Residential urban greenspace (through NDVI, land use cover, and amount of tree cover	108	respiratory health - respiratory mortality, lung cancer, respiratory hospital visits, asthma, lung function, rhinitis	Children and adults	N/A	In 26 countries across Europe, the Americas, and Asia	Risk of bias, and overall quality of evidence assessed using the Navigation Guide methodology and criteria	N/A	The quality of studies related to respiratory mortality were of moderate quality, while for the other outcomes were rated as low.	Overall, the strongest evidence of a positive association between greenspace and health related to respiratory mortality. Only 7 out of 20 studies were rated as probably low rating of bias. Moreover, although asthma and greenspace association was the most studied (38 studies), the results were too inconsistent.	It was estimated that per 0.1 unit increase in residential NDVI levels of greenspace was associated with a 3-6% lower risk of respiratory mortality. Moreover, the amount of residential green space may linked to higher smoking cessation, providing a pathway for reducing lung cancer risk. The evidence regarding asthma and green space exposure was to heterogeneous and inconsistent.
The effect of greenness on allergic rhintis outcomes in children and adolescents: a systematic review and meta-analysis	Cao et al. (2023)	14, and 11 for the meta-analysis	N/A	Grenness	NDVI (n=12) and others (n=2)	AR	Age ranged from 0-17 yr	Ranged from 522 to 642,313	Europe (n=9), China (n=3), and USA (n=1), and multicentric (n=1)	RoB with NTP/OHAT	OR with 95%CI was the unified effect estimates for the meta-analysis. **No significant effects of greenness exposure on AR on the meta-analysis**.	Overall, 7 were classified as Tier 1, and the rest as Tier 2 for RoB.	Very few studies and high heterogeneity in the assessment of exposure.	Some studies found **no significant association** between grenness at different buffer sizes and **AR** (n=4), another found a significant increases in the risk of AR (n=1). Overall, this review failed to find significant associatios between greenness exposure and AR.
Association between greenspace and blood pressure: a systematic review and meta-analysis	Zhao, et al. (2022)	38	N/A	Greenspace exposure (through NDVI, land use/cover, proportion of green space, distance to green space)	38	Cardiovascula r health- hypertension and BP levels	Children and adults	Sample ranged from 73 to over 3.9 million participants, with a total approximately of 5.2 million	Europe (n=16), América (n=11), Asia (n=9), and Australia (n=2)	Quality assessed by the NOS scale (score 0-9), risk of bias using the OHAT tool. Meta-analysis results reported as odds ratio with 95% confidence interval.	N/A	25 studies rated as high quality, and none were excluded for high risk of bias	N/A	15 out of 23 studies found a significantly beneficial effect for increasing NDVI green space and BP levels or prevalence of hypertension.
Psychosocial and physiological health outcomes of green exercise in children and adolescents: a systematic review	Mnich, et al. (2019)	14	2008-2019	Green exercise (physical exercise in green spaces)	14	Psychological and physiological outcomes- self-esteem, BP	Age: 6 to 17 y/o	a total of 9402	the UK (n=5), the US (n=5), Australia (n=2), and Japan	Risk of bias and quality of study was assessed using the Effective Public Health Practice Project (EPHPP) tool	N/A	Except for one study that was rated as moderate quality, all were reported as low quality. Due to selection bias, blinding, collection methods.	No meta-analysis conducted due to high heterogeneity in the health outcomes measured.	PA in green spaces showed stronger positive effects in attention and antisocial , and higher health-related quality of life than doing PA in other environments. For other several outcomes, PA did not have an effect in any condition or was not different between green and control condition. For systolic BP, contradictory results were found.
Nature and children's health: a systematic review	Song et al. (2021)	296	N/A	Nature exposure	Residential green space (n=147), general green space activity (n=35), School green space (n=40)	PA (n= 108), cognitive behavioural and mental health (n=85), BMI (n=45), cardiovascular and metabolic measures, asthma and allergy, academic and learning	0-18 yr	N/A	N/A	Mixed Methods Appraisal Tool (MMAT) for quality evidence and Risk of Bias in Non-randomized studies of interventinos (ROBINS-I)	N/A	Cross-sectional studies had low RoB, experimental studies were at high RoB because of participants not being representative of target population, and randomized-controlled trials had a high RoB. Studies on BMI were considered as moderate.	Wide variability in participant samples, nature exposures, and outcome measures. Sample sizes and geographic locations are not displayed.	**PA**: most studies (n=71) reported positive associations, and 3 reported negative ones. **Cognitive, behavioural, and mental health**: most found positive associations (n=71), with 2 reporting negative associations with green nature exposure. Attention and depression were the most common outcomes. The 5 randomized-controlled trials reported positive associations with well-being, mental fatigue, emotional status, perceived stress adn restorativeness. **BMI**: Around one third (n=16) reported positive associations, but more than half (n=27) had mixed or null results. **Allergy and asthma**: 13 studies reported positive associations, negative associations were reported in 8. **Cardiovascular & metabolic outcomes**: 7 studies reported positive associations and 9 reported null or mixed. Overall, most negative associations were for asthma and allergy outcomes, which had considerable heterogeneity in diagnosis and type of allergen
"Kids get in shape with nature": a systematic review exploring the impact of green spaces on childhood obesity	Alejandre, et al. (2020)	16	N/A	Green and blue space exposure	16	Physical activity (by intensity, frequency, duration, and distance, and/or proportion of children engaging in intensive PA)	Age: 5 to 18 y/o	NR	New Zealand, UK, US, The Netherlands, Canada, Turkey, and Germany	Quality assessment using the NHLBI and CASP tools (score: 0-12)	N/A	N/A	N/A	Playing and performing other activities in natural environments such as nature reserves, sport fields, or playgrounds result in children engaging to longer moderate to vigorous PA, as well as has a positive effect on their eating behaviours. Five studies demonstrated that proximity and increased availability to green spaces in the neighbourhood or near the residential home increase engagement of children into moderate to vigorous PA. Indeed, after participating in school vegetable gardens, children show more enthusiasm, ownership and involvement in vegetable production and consumption. Green space use is influenced by demographic aspects such as ethnicity, gender, social and parental factors. Girls are more associated with low engagement and low level of PA in green spaces, while boys engage more in moderate to vigorous PA when the environments are wider.
Growing up green: a systematic review of the influence of green space on youth development and health outcomes	Sprague, N. et al. (2022)	28	2014-2020	Green space exposure, measured either through distance to greenspace, use of greenspace and frequency of exposure by NDVI measurements	28	Cognitive health- mental health, brain development, attention and behaviour. Respiratory and cardiovascular health	Children and adolescents: 2-18 years old	N/A	Canada, USA, Spain, France, UK, Germany	Quality assessed through a 10-item quality assessment checklist, adapted from a previous review on green space exposure and obesity.	N/A	Overall quality was moderated, does not give exact value for each study	Inconsistent measures of greenspace and cofounders, inadequate	Half of the studies (n=3) on cognitive and brain development found no associations, while the other half found positive health associations (n=3). Green exposure was associated with increased working memory, cognitive performance, and white and grey matter volume in several regions of the brain. All studies evaluating mental health and green exposure found positive health associations (n=13). It was associated with improved mood, emotional resilience, increased happiness, improved prosocial behaviour, and decrease risk of developing schizophrenia. It was also linked with improved attention, improved self-determination, and lower risk for ADHD. Moreover, living near green spaces was associated with lower prevalence of asthma and allergic rhinitis, and lower odds of overweight status.
Systematic review: neurodevelopmental benefits of active/passive school exposure to green and/or blue spaces in children and adolescents	Díaz-Martínez et al. (2023)	28	2017-2022	Green and blue spaces	Availability of surrounding greennery; accessibility to green and/or blue spaces; other indicators related to natural spaces; and active interventions	Neurodevelopment/ neurobehaviour: cognitive/academic performance (15), neurodevelopmental disorders and diseases (n=4), working memory (n=3), [illegible]	School-children and adolescents	494,963- from 25 to 344,175	USA (n=11), Europe (n=11), China (n=2), Brazil (n=1), Australia (n=2), Canada (n=1),	QuADS	N/A	Total score of the studies was high, with an average score of 32.4 and a maximum of 39.	High heterogeneity of the methodologies of the studies included, and the existing research requires more cofounding factors	Grenness was positively associated with academic performance (n=8), one found a negative significant association. Regarding blue exposure, the two studies did not find a significant association, and only one found a positive one. Five studies found that exposure to natural environments and activities with natural elements within the classroom can positively affect attention control. Two out of three found positive effects of nature on working memory. Three out of three found positive associations between student's emotional well-being and greenness. 4 out of 8 found an improvement in neurobehaviour, and the remaining found null associations. Surrounding greenness was related to improvements in behavior and self-regulation (n=2), and regulation of compulsive disorders (n=1). 2 studies found beneficial effecs on autism, and one found significant association with lower odds of ADHD, and a decrease in problem behaviours (n=1).

The effects of park-based interventions on health-related outcomes among youth: a systematic review	Wallace, et al. (2022)	15	N/A	Green and blue space exposure interventions	15	Health outcomes, such as PA, BMI, or blood pressure	Mean ages were 8 and 13 y/o	Samples ranged from 39 to 2464 children	USA (12), Australia, Denmark, and The Netherlands	Quality assessed using 5 domains: description of the study, of the sampling, measurement and scale information, data analysis information and interpretation of results	N/A	Most studies had an average of medium to high study quality	Number of relevant studies was limited	They found that interventions in public parks can result in improvements in health-related outcomes in children and adolescents. All identified interventions were positively associated with individual-level and park-level outcomes ranging from body weight, moderate-to-vigorous-intensity physical activity, park utilization, and health behaviour knowledge.
Outdoor blue spaces, human health and well-being: a systematic review of quantitative studies	Gascón, et al. (2017)	35	N/A	Outdoor blue exposure (measured at blue exposure at residence, except 3 that measured blue exposure in general, and 2 that measured around school location)	35	General health, mental health and well-being, physical activity, obesity and CV outcomes	Adults, adolescents, and schoolchildren	N/A	UK (11), Croatia (3), Spain (2), France (2), The Netherlands (1), Finland (1), Ireland (1), Australia (6), China (3), New Zealand (2), Canada (1), USA (1), and Yemen (1).	The quality evaluation was using an adapted version of the criteria used in a previous review on green spaces by Gascon et al. (2016)	N/A	22 out of 35 studies were rated as good quality, with 1 of excellent quality, 6 rated as fair quality, and remaining were rated of poor quality	The number of studies focusing on children and adolescents was very limited, with only 8 studies investigating this population. The outcomes were measured with self-reporting, which can be prone to bias.	Exposure to blue spaces increased emotional well-being in children 11-16 years old. Moreover, parents spending more time at the beach was associated with children reporting less emotional problems, and more prosocial behavior, but no association with ADHD. Adolescents spending more time at the beach showed better recalled restoration than urban green spaces, they felt more relaxed. Children and adolescents living more far away from the beach reported higher BMI than those living nearer in two studies. Also, those adolescents that exercised in the beach as they lived near were more likely to meet the physical activity guidelines in winter and summer than those more than 800m from the beach.
